# Coexistence of Metallocene Cations and Anions

**DOI:** 10.1021/jacs.5c09718

**Published:** 2025-09-15

**Authors:** Nico Gino Kub, Robin Sievers, Marc Reimann, Tim-Niclas Streit, Simon Steinhauer, Johanna Schlögl, Martin Kaupp, Moritz Malischewski

**Affiliations:** † Institut für Chemie und Biochemie − Anorganische Chemie, 9166 Freie Universität Berlin, Fabeckstr. 34-36, Berlin 14195, Germany; ‡ Institut für Chemie, 26524 Technische Universität Berlin, Straße des 17. Juni 135, Berlin 10623, Germany; § Institut für Ionenphysik und Angewandte Physik, Universität Innsbruck, Innsbruck A-6020, Austria

## Abstract

We report the synthesis and structural characterization of the rhodocene anion [Rh­(C_5_Me_5_)­(C_5_(CF_3_)_5_)]^−^ [**1**]^−^ as the [Co­(C_5_Me_5_)_2_]^+^ salt, representing an unprecedented coexistence of metallocene cations and anions in different oxidation states. [Co­(C_5_Me_5_)_2_]^+^ [**1**]^−^ was synthesized by the reduction of the rhodocenium cation [Rh­(C_5_Me_5_)­(C_5_(CF_3_)_5_)]­[BF_4_] ([**1**]^+^[BF_4_]^−^) with two equivalents of decamethylcobaltocene [Co­(C_5_Me_5_)_2_], since the strongly electron-withdrawing effect of the [C_5_(CF_3_)_5_]^−^ ligand shifts the first and second reduction potentials of [**1**]^+^ to moderate values of −0.90 and −1.46 V vs FcH^0/+^. [Co­(C_5_Me_5_)_2_]^+^[**1**]^−^ was characterized by single-crystal X-ray diffraction (XRD), providing the first example of a structurally characterized 4d metallocene anion. Whereas the Rh^III^ cation has two η^5^-bound Cp ligands, the perfluorinated Cp* ligand is only η^3^-bound in the Rh^I^ anion in order to obey the 18-electron rule. The reduction of the rhodocenium center is accompanied by a significant shift of the ^103^Rh NMR signal from −9308 ppm of [**1**]^+^ to −6895 ppm of [**1**]^−^ (referenced to Rh­(acetylacetonate)_3_).

## Graphical abstract

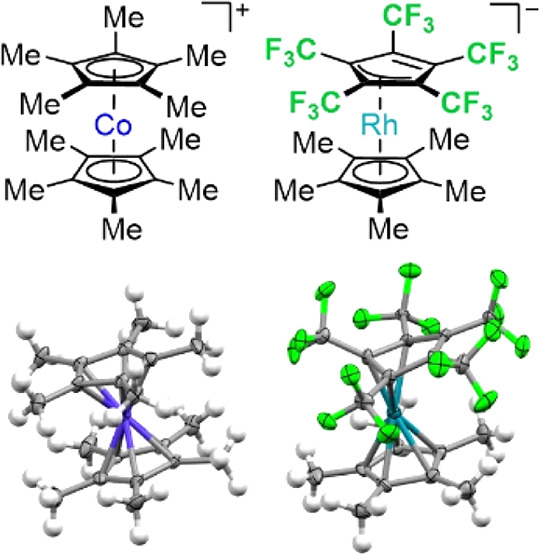

## Introduction

The synthesis and characterization of ferrocene (FcH) in 1951 have been widely marked as the birth of organometallic chemistry, sparking the rich chemistry of metallocenes.^1^ Ever since, the continuous improvement of synthetic possibilities has allowed for the isolation and characterization of unique cyclopentadienyl compounds,^2^ or especially sandwich compounds,^3^ ranging from main group^4^ to transition metal chemistry,^5^ as well as highly radioactive elements.^6^ The rich synthetic chemistry of ferrocenes allows even most elaborate molecular architectures^7,8^ but also provides the necessary flexibility for functionalization reactions with regard to applications, e.g., for medicinal chemistry,^9^ battery design,^8^ atomic sensors,^10^ and catalysis.^11^ A defining feature of metallocenes is their unmatched redox chemistry, with the one-electron oxidation of ferrocene to ferrocenium serving as a standard reference for nonaqueous electrochemical processes.^12^ Considering that transition metal metallocenes usually traverse between formal +II and +III oxidation states, corresponding to the neutral metallocene [MCp_2_] (M = metal, Cp = cyclopentadienyl) and cationic metallocenium [MCp_2_]^+^, the further expansion of these boundaries has gained a lot of attention in recent years.^13^ The synthesis and characterization of the nickelocenium dication^14^ and decamethylferrocenium dication^15^ mark recent advances in the oxidation of metallocenes to the formal +IV metal oxidation state (Figure 1).

**Figure 1:**
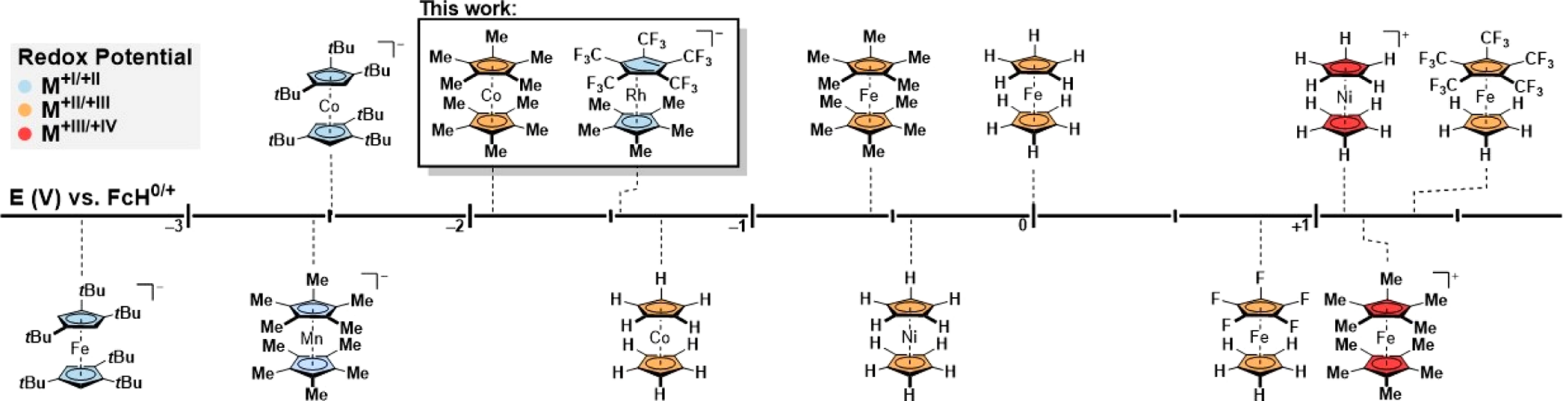
Experimentally obtained half-wave redox potentials (*E*_1/2_) in V vs FcH^0/+^ of various metallocene anions,^18,19,23^ neutral metallocenes,^14,21,24,25^ and metallocene cations.^14,15^

Compared to the synthesis of metallocene cations, metallocene anions [MCp_2_]^−^ have remained far less explored with only a handful of synthetically isolated compounds.^16–20^ As metallocene anions require a formal +I metal oxidation state, group 1 metallocenes represent the simplest metallocene anions. Since the +I oxidation state is most stable for alkali metals, the synthesis of group 1 metallocene anions is not dependent on any reducing agents or bulky Cp substituents.^20^ In contrast, the unstable nature of d-block metallocene anions demands not only harsh reducing agents for their synthesis but also the stabilization of these highly reactive anionic species. Recent approaches, such as the isolation of the derivatized manganocene, ferrocene, and cobaltocene anions,^19^ have demonstrated the efficiency of bulky substituents in stabilizing the reactive metal center. As harsh reductive conditions are required to generate metallocene anions, inert spectator cations are a necessity. Thus far, alkali metal cations have been the sole choice, as their reduction potentials surpass those of the corresponding metallocene anions.^16^ In principle, other cations could also be suitable, given that their reduction potential transcends that of the corresponding metallocene anion. While this has never been realized, the substantial effect of Cp substitution patterns on the redox properties of metallocenes could in principle allow metallocene cations and anions to coexist in one compound. As decamethylcobaltocenium [Co­(C_5_Me_5_)_2_]^+^ has one of the lowest reduction potentials (*E*_1/2_ = −1.94 V, in CH_2_Cl_2_ vs FcH^0/+^) of a metallocene cation, the corresponding metallocene anion would need to exceed this reduction potential to a sufficient extent to prevent an electron transfer, which results in two neutral metallocenes.^21,22^

While not yet investigated in this context, the introduction of fluorinated electron-withdrawing substituents into the Cp scaffold offers a novel approach toward isolating metallocene anions with dramatically increased redox potentials. This has been demonstrated exemplarily for ferrocenes, where the addition of every fluorine substituent linearly increases the redox potentials, e.g., *E*_1/2_ = +0.14 V (in CH_2_Cl_2_ vs FcH^0/+^) for monofluoroferrocene and *E*_1/2_ = +0.82 V (in CH_2_Cl_2_ vs FcH^0/+^) for pentafluoroferrocene [Fe­(C_5_H_5_)­(C_5_F_5_)].^24,26^ This effect is substantially more pronounced for trifluoromethyl groups (CF_3_) due to the absence of conjugative donor effects, with two CF_3_ substituents increasing the redox potential to a staggering *E*_1/2_ = +0.64 V (in CH_2_Cl_2_ vs FcH^0/+^).^27,28^ Additionally, CF_3_ groups also offer greater steric shielding compared to fluorine substituents.

The first synthesis of the perfluorinated Cp* was described in 1980 by Lemal et al. and later improved by Chambers et al.^29^ In previous works, we have demonstrated the first coordination of [C_5_(CF_3_)_5_]^−^ and its unique reactivity toward substitution lability, as well as the increased oxidative stability compared to ordinary Cp ligands.^29,30^ Recently, we have shown that the substitution of one [C_5_H_5_]^−^ ligand in ferrocene by [C_5_(CF_3_)_5_]^−^ shifts the half-wave potential up to *E*_1/2_ = +1.35 V (in hexafluoroisopropanol vs FcH^0/+^).^25^ Since common metallocene anions are formed under strongly reducing conditions (*E* < −2.5 V vs FcH^0/+^, see Figure 1), metallocenes bearing [C_5_(CF_3_)_5_]^−^ as a ligand might enable the synthesis of a metallocene anion with a redox potential significantly more positive than −2 V, thereby potentially allowing the synthesis and isolation of a metallocene anion paired with decamethylcobaltocenium [Co­(C_5_Me_5_)_2_]^+^ as a counterion.^21^

## Results and Discussion

The synthesis of the mixed rhodocenium [Rh­(C_5_Me_5_)­(C_5_(CF_3_)_5_)]­[BF_4_] (hereinafter referred to as [**1**]^+^[BF_4_]^−^) was achieved in analogy to literature-known routes^31^ by the reaction of commercially available [Rh­(C_5_Me_5_)­Cl_2_]_2_ and [Ag]­[BF_4_] in the presence of [NEt_4_]­[C_5_(CF_3_)_5_], which occurs presumably via the *in situ* formation of the solvated 12-electron fragment [Rh­(C_5_Me_5_)]^2+^. After purification, [**1**]^+^[BF_4_]^−^ was isolated with an overall yield of 49% and represents the first rhodocenium species with a [C_5_(CF_3_)_5_]^−^ ligand (Figure 2A).

**Figure 2:**
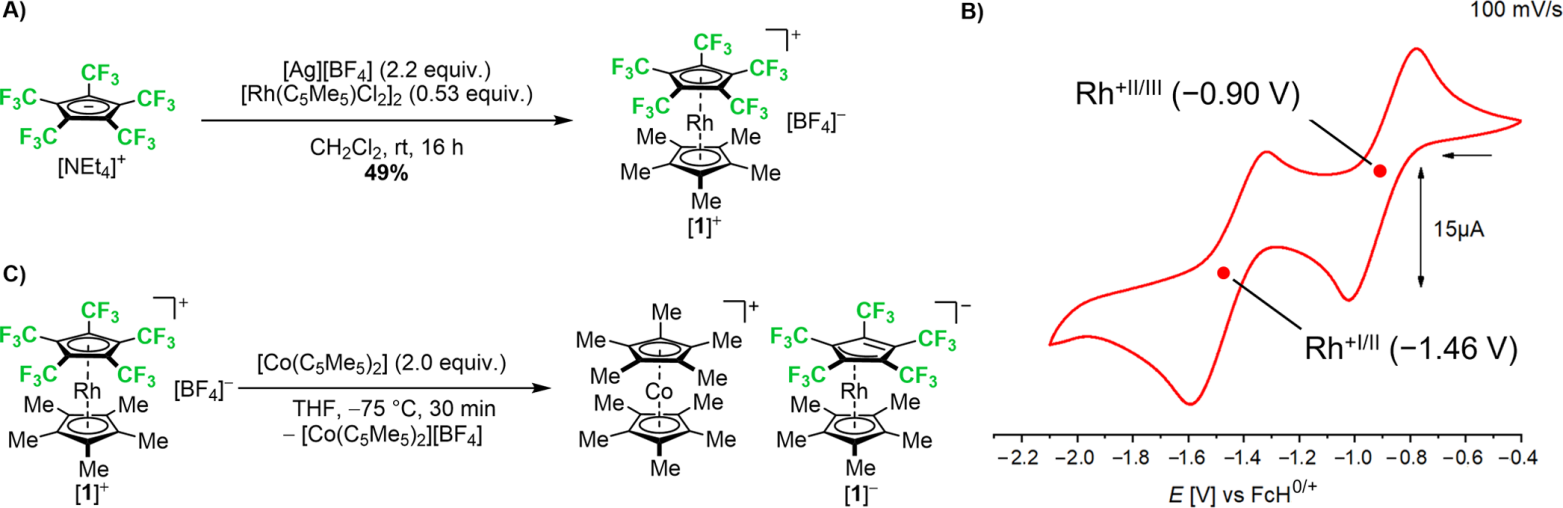
(A) Synthesis of [Rh­(C_5_Me_5_)­(C_5_(CF_3_)_5_)]­[BF_4_] from [NEt_4_]­[C_5_(CF_3_)_5_] and [Rh­(C_5_Me_5_)­Cl_2_]_2_. (B) Cyclic voltammogram (current in μA vs potential in V vs FcH^0/+^; scan rate: 100 mV/s) of [Rh­(C_5_Me_5_)­(C_5_(CF_3_)_5_)]­[BF_4_] (14 μmol) in anhydrous degassed THF with 0.1 M [nBu_4_N]­[PF_6_] as an electrolyte and a platinum working electrode. (C) Reduction of [**1**]^+^[BF_4_]^−^ with [Co­(C_5_Me_5_)_2_] toward [Co­(C_5_Me_5_)_2_]^+^[**1**]^−^.

The ^19^F NMR spectrum revealed a singlet at δ = −51.8 ppm (in CD_2_Cl_2_), which is somewhat high-field shifted compared to ionic [C_5_(CF_3_)_5_]^−^ (δ = −50.5 ppm in CD_2_Cl_2_). The ^1^H NMR revealed a singlet at δ = 2.14 ppm (in CD_2_Cl_2_) for the [C_5_Me_5_]^−^ coligand. Additionally, a rhodium shift was observed in the ^1^H, ^103^Rh HMQC spectrum at δ = −9308 ppm (in CD_2_Cl_2_, referenced to Rh­(acetylacetonate)_3_, Figure S5).

Single crystals of [**1**]^+^[BF_4_]^−^ were obtained by slowly cooling a solution of the respective compound in a mixture of CH_2_Cl_2_ and *n*-pentane to −75 °C, crystallizing in the triclinic space group *P*1̅. The asymmetric unit with two [**1**]^+^[BF_4_]^−^ moieties revealed an η^5^-coordination of both the [C_5_(CF_3_)_5_]^−^ and [C_5_Me_5_]^−^ ligands (Figure S11). Interestingly, the averaged Rh–C_Cp_ bond length for [C_5_(CF_3_)_5_]^−^ (2.222(8) Å) and Rh–Cp_centroid_ distance (1.854(1) Å) are slightly larger compared to the averaged Rh–C_Cp_ bond length for [C_5_Me_5_]^−^ (2.174(9) Å) and Rh–Cp_centroid_ distance (1.810(1) Å), while the C_Cp_–C_Cp_ distances are relatively similar for both Cp ligands. These findings are also reproduced in quantum chemical calculations at the r^2^SCAN-3c level, revealing averaged Rh–C_Cp_ bond lengths for [C_5_(CF_3_)_5_]^−^ (2.245 Å)/[C_5_Me_5_]^−^ (2.194 Å) and Rh–Cp_centroid_ distances of 1.882 and 1.819 Å, respectively. Surprisingly, these observations are in contrast to the recently reported structures of [Fe­(C_5_H_5_)­(C_5_(CF_3_)_5_)][Bibr ref25] and [Ru­(C_5_H_5_)­(C_5_(CF_3_)_5_)].^32^ For the former, the Fe–Cp_centroid_ distances were 0.05 Å shorter for the perfluorinated ligand. In the case of ruthenocene, the same trend (but with differences of less than 0.01 Å) was found. Both observations were in agreement with the quantum chemical calculations. To date, the reasons behind these structural differences are not fully understood. It could either be a consequence of stronger π-donation of the [C_5_Me_5_]^−^ ligand in the case of Rh­(III) or result from steric repulsion between the [C_5_Me_5_]^−^ and the [C_5_(CF_3_)_5_]^−^ ligands, which pushes the weaker-bound perfluorinated ligand away from the metal center.

To determine the reduction potentials to the corresponding rhodocene [**1**] and rhodocene anion [**1**]^−^, a cyclic voltammetry study was performed in a tetrahydrofuran (THF) solution of [**1**]^+^[BF_4_]^−^ at room temperature, with [*n*Bu_4_N]­[PF_6_] as the supporting electrolyte (Figure 2B). A quasi-reversible reduction process was observed at *E*_1/2_ = −0.90 V (vs FcH^0/+^), which likely corresponds to the Rh^III^/Rh^II^ one-electron reduction toward the neutral rhodocene (see also Figure S16 and Table S13). For further investigations, [**1**]^+^[BF_4_]^−^ was reduced with [Co­(C_5_H_5_)_2_] in THF at −20 °C, which led to the immediate formation of a dark blue suspension. The neutral rhodocene [**1**] was investigated via EPR spectroscopy. The X-band EPR spectrum of the neutral rhodocene [**1**] in THF at −196 °C shows three partially overlapping features, suggesting a rhombic *g*-tensor (*g*_*x*_ = 1.877, *g*_*y*_ = 1.998, *g*_*z*_ = 2.085). This aligns well with the spectrum observed for [Rh­(C_5_HPh_4_)_2_] but is in contrast to the axial spectrum reported for [Rh­(C_5_H_5_)_2_].^33^ Our findings for the fluorinated rhodocene [**1**] also agree very well with quantum chemically obtained values (*g*_*x*_ = 1.846, *g*_*y*_ = 1.998, *g*_*z*_ = 2.084) (Figure S14). Due to solubility issues and decomposition at temperatures above −20 °C, it was not possible to obtain single crystals of [**1**]. Quantum chemical calculations at the r^2^SCAN-3c level predict an η^4^-coordination mode of the [C_5_(CF_3_)_5_]^−^ ligand with four Rh–C distances in the range of 2.158–2.295 Å and one longer distance of 2.566 Å.

A second quasi-reversible reduction process was observed at *E*_1/2_ = −1.46 V (vs FcH^0/+^) and likely corresponds to the Rh^II^/Rh^I^ one-electron reduction toward the rhodocene anion. Compared to other substituted rhodocenium derivatives, [**1**]^+^[BF_4_]^−^ revealed substantial increases of redox potentials of up to 1.0 V for both the first and second reductions.^34–36^

Similar to the introduction of an indenyl ligand [C_9_H_7_]^−^, the [C_5_(CF_3_)_5_]^−^ ligand enhances the stability of the neutral and anionic rhodocene as indicated by increased redox potentials, resulting from a strong electron-withdrawing effect, as well as a facilitated η^5^/η^3^-ring slippage, which results in an 18-electron rather than a 20-electron complex.^36^ Based on the observed reduction potential toward the rhodocene anion [**1**]^−^ and the relative position toward the oxidation potential of decamethylcobaltocene [Co­(C_5_Me_5_)_2_], the latter was assumed to be a suitable reducing agent. Consequently, the 2-fold reduction of [**1**]^+^[BF_4_]^−^ with [Co­(C_5_Me_5_)_2_] in THF at −75 °C was carried out, resulting in the formation of [Co­(C_5_Me_5_)_2_]^+^[**1**]^−^, which marks the first instance of metallocene cations and anions in different oxidation states coexisting in one compound (Figure 2C). As the reduction chemistry of rhodocene cations is notoriously challenging,^35,36^ the synthesized [Co­(C_5_Me_5_)_2_]^+^[**1**]^−^ represents the first isolated and structurally characterized rhodocene anion and also the first 4d metallocene anion to date.

The ^19^F NMR spectrum at −80 °C in *d* ^8^-THF revealed three singlet signals at δ = −49.3, −53.3, and −58.1 ppm, indicating an η^3^-coordination of [C_5_(CF_3_)_5_]^−^, where the individual positions seem to be locked, or the exchange is slower than the NMR time scale. Slow decomposition was already observed starting at −70 °C, further supporting the temperature sensitivity of the rhodocene anion. Furthermore, a rhodium shift was observed in the ^1^H, ^103^Rh HMQC spectrum at δ = −6895 ppm (in *d* ^8^-THF, vs Rh­(acetylacetonate)_3_, Figure S10), which is 2413 ppm more positive than the shift of the corresponding cationic Rh^III^ compound (−9308 ppm). Quantum chemical calculations of the ^103^Rh shifts at the quasirelativistic two-component ZORA level (see Supporting Information) find excellent agreement for both species (−6610 and −9570 ppm, respectively). Additional calculations of the shift for the Rh^III^ species at the structure of the Rh^I^ species find −7090 ppm, suggesting that the less negative value results predominantly from the lower coordination number in the Rh^I^ complex. To verify the description of [**1**]^−^ as Rh^I^ and to rule out ligand-based reductions, we have analyzed the wave function using natural population analysis (NPA)^37^ and quantum theory of atoms in molecules (QTAIM).^38^ Reduction of [**1**]^+^ to [**1**]^−^ decreases the charges associated with the Rh center, as well as increases the size of the rhodium atom (defined by the volume of the metal attractor basin in the QTAIM analysis), both of which are concurrent with the reduction from Rh^III^ to Rh^I^. Further comparison with [Rh^III^(C_5_H_5_)_2_]^+^ and [Rh^I^(C_5_Me_5_)­(CO)_2_] also supports this assignment (see Table S10 for details).

For further structural confirmation, single crystals of [Co­(C_5_Me_5_)_2_]^+^[**1**]^−^ were obtained by layering a saturated THF solution with *n*-hexane at −75 °C. The respective salt crystallized in the monoclinic space group *P*2_1_/*n*, with one [Co­(C_5_Me_5_)_2_]^+^[**1**]^−^ fragment in the asymmetric unit (Figure 3, left). The Co–C_Cp_ averaged bond length of 2.047(7) Å, as well as the Co–Cp_centroid_ distances of 1.645(1) and 1.649(1) Å correspond to published Co–C_Cp_ bond lengths of other [Co­(C_5_Me_5_)_2_]^+^ compounds and are significantly shorter than the Co–C_Cp_ averaged bond length of 2.099(9) Å and Co–Cp_centroid_ distances of [Co­(C_5_Me_5_)_2_], confirming the cationic nature of the cobaltocenium moiety in [Co­(C_5_Me_5_)_2_]^+^[**1**]^−^.^39^ The η^3^-coordination of the [C_5_(CF_3_)_5_]^−^ ligand in [**1**]^−^ is the most notable. It is perfectly reproduced in quantum chemical calculations at the r^2^SCAN-3c level. As the reduced π-donor ability of [C_5_(CF_3_)_5_]^−^ coincides with smaller M–Cp interaction energies,^27^ which facilitates an η^5^/η^3^ ring slippage, the η^3^-coordination effectively reduces the Pauli repulsion between the Rh^I^ center and the [C_5_(CF_3_)_5_]^−^ ring originating from the population of antibonding orbitals upon reduction (see Table S11) and thereby stabilizes the anionic species. This also lowers the symmetry of the system, avoiding a low-spin 20-electron metallocene and resulting in a more stable 18-electron system. While the terminal positions (C2, C5) of the [C_5_(CF_3_)_5_]^−^ allylic binding motif exhibit an average Rh–C_Cp_ bond length of 2.198(7) Å, the central position (C1) has a significantly shortened Rh–C_Cp_ bond length of 2.007(7) Å (Figure 3). These substantially divergent bond lengths are a consequence of the ring slippage, moving the center of the Cp ligand away from the metal center and thereby shortening the distance toward the central allylic position. Consequently, the adjacent CF_3_ substituent is significantly bent out of plane, with an angle of 24.09(66)°, and the [C_5_Me_5_]^−^ coligand is tilted out of plane relative to the allylic system (6.89(50)°), minimizing the steric clash between adjacent CH_3_ and CF_3_ substituents (Figure S12). Furthermore, the uncoordinated C3=C4 bond is bent out of the plane formed by the allylically bound C5–C1–C2 moiety at an angle of 23.94(56)° (Figure S13). The C1–C2 and C1–C5 bond lengths of 1.482(9) and 1.476(9) Å, respectively, only differ by 0.006 Å, which falls within the error intervals of both bonds, indicating a complete electron delocalization across the allylic system. As a consequence of the allylic structure, the C3–C4 bond length of the unbound positions (1.355(10) Å) is severely shortened and close to the bond length of unconjugated C=C double bonds.^40^

**Figure 3:**
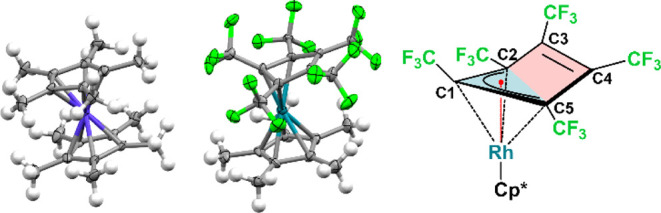
Left: molecular structure in the solid state of [Co­(C_5_Me_5_)_2_]­[Rh­(C_5_Me_5_)­(C_5_(CF_3_)_5_)]. Ellipsoids are depicted with 50% probability level. Color code: gray-carbon; green-fluorine; blue-cobalt; turquoise-rhodium. Right: drawing of the allylic-bound [C_5_(CF_3_)_5_]^−^ ligand toward rhodium depicting the C1-bound CF_3_ substituent, as well as the unbound C3–C4 double bond tilted out of plane.

We further analyzed the reduction of [**1**]^+^ to [**1**]^−^ quantum chemically by means of energy decomposition analysis (see Table S11). Adding two electrons to [**1**]^+^ populates the e*_1g_ orbitals (in D_5d_ notation) of the metallocene. This reduces the π-bonding interaction between the ring and metal center by half (lowering Δ*E*_Orb.Int_, see Tables S11 and S12) and increases Pauli repulsion (Δ*E*_Pauli_), leading to a significantly reduced interaction energy (Δ*E*_Int_). Slippage of the ring then results in both a symmetry reduction (avoiding near-degeneracies in the overall singlet state) and a reduction in Pauli repulsion. At the same time, the orbital interaction is increased significantly. This can be traced back to a strong π-back-bonding interaction from the Rh^I^ center to the LUMO of the slipped [C_5_(CF_3_)_5_]^−^ ring (see Table S12), which is located mostly at the allylic part and is completely absent in [**1**]^+^. The interaction is enhanced by a sizable decrease of the LUMO energy (from −0.59 to −2.23 eV) upon distortion. As a result of the structural relaxation, the overall computed interaction energy with the [C_5_(CF_3_)_5_]^−^ ligand becomes very similar for the Rh^III^ and Rh^I^ complexes.

## Conclusion

The significant electron-withdrawing power of the [C_5_(CF_3_)_5_]^−^ ligand and its ability to dramatically shift the redox potential of metallocene anions to less negative values enabled the first demonstration of the coexistence of metallocene cations [Co­(C_5_Me_5_)_2_]^+^ and anions [Rh­(C_5_Me_5_)­(C_5_(CF_3_)_5_)]^−^ [**1**]^−^ in different oxidation states in one compound. [**1**]^−^ constitutes the first structurally characterized 4d metallocene anion, further adding to the scarce chemistry of the d-block metallocene anions. The reduced interaction energy of [C_5_(CF_3_)_5_]^−^ toward Rh facilitates η^5^/η^3^ ring slippage and thereby stabilizes the anionic species by reducing the electron count from 20 to 18 for the Rh^I^ center. Hereby, the η^3^ structure of the anionic species not only decreases Pauli repulsion but also increases back-bonding interactions, thereby stabilizing the anion in two distinct ways. Compared to the cationic rhodocenium species [**1**]^+^, reduction from Rh^III^ to Rh^I^ shifts the ^103^Rh signal from −9308 to −6895 ppm (vs Rh­(acetylacetonate)_3_). Quantum chemical calculations have shown that the ^103^Rh chemical shift of [**1**]^+^ and [**1**]^−^ is largely influenced by the change in coordination arrangement rather than the change in oxidation state.

## Supplementary Material



## Notes

Experimental details, spectroscopic, structural, and computational data (PDF)

## #

M.R. Universität Innsbruck, Institut für Ionenphysik and Angewandte Physik, A-6020 Innsbruck, Austria

## Notes

A preprint version of this work was deposited at ChemRxiv.^41^
