# Supplementary material for: Coexistence of Metallocene Cations and Anions
Source: J Am Chem Soc. 2025 Sep 15;147(38):34641–6. doi: 10.1021/jacs.5c09718 (PMC12465000; doi:10.1021/jacs.5c09718)
Supplement: Supplementary file 1 [file ja5c09718_si_001.pdf]

Supporting Information

Coexistence of Metallocene Cations and Anions

Nico Gino Kub, Robin Sievers, Marc Reimann, Tim-Niclas Streit, Simon Steinhauer, Johanna Schlögl, Martin Kaupp, Moritz Malischewski*

Author affiliations:

N. G. Kub, R. Sievers, T.-N. Streit, S. Steinhauer, J. Schlögl, M. Malischewski: Freie Universität Berlin, Institut für Chemie und Biochemie – Anorganische Chemie, Fabeckstr. 34-36, 14195 Berlin, Germany.

E-Mail: moritz.malischewski@fu-berlin.de

M. Reimann, M. Kaupp: Technische Universität Berlin, Institut für Chemie, Straße des 17. Juni 135, 10623 Berlin, Germany

M. Reimann (current address): Universität Innsbruck, Institut für Ionenphysik und Angewandte Physik, A-6020 Innsbruck, Austria

General Information

Unless otherwise noted, all reaction and workups were performed open to air. Reactions and workups involving substances sensitive to oxygen and water were performed in previously heated glassware under an atmosphere of argon using standard Schlenk techniques and an oil pump vacuum of 10^{-3} mbar. Room temperature (rt) refers to 20 °C. The addition of liquid reagents and solvents was done by using threefold argon-flushed disposable syringes and septa, while solids were added in argon stream. Low temperature reactions were performed in an ice- respectively dry ice/ethanol-bath. Glassware was cleaned by storing in a potassium hydroxide bath for several days, rinsed with diluted hydrochloric acid and doubly deionized water and dried at 150 °C.

Pressure Reactions

The synthesis of $[NEt_4][C_5(CF_3)_5]$ involves high temperatures and highly volatile substances in a closed system and was performed in specially suited glassware. Hence, it must be assumed, that high pressures arise upon heating and advanced caution is required. Therefore, it is advisable to perform the reaction in a separate and properly closed fume hood. The reaction vessel should not be opened and if possible, not even touched until the reaction has finished and reached rt.

Solvents and reagents

Anhydrous *n*-pentane, CH_2Cl_2 and MeCN were obtained from the solvent system FMBRAUN MB SPS800 and stored over activated 3 Å molecular sieves. *N*-hexane was used as purchased and stored over activated 3 Å molecular sieves. Et_2O and *n*-Bu_2O were used as purchased. Similarly, deuterated solvents CD_2Cl_2 and *d*^{8}-THF were used as purchased and stored over activated 3 Å molecular sieves. THF was distilled from molten potassium. Sulfolane was heated at 60 °C for at least 24 h over activated 3 Å molecular sieves and additionally 1 h in high vacuum prior to use. 18-Crown-6 was heated at 80 °C for at least 2 h in high vacuum prior to use. Water was doubly deionized. All other solvents and commercially available reagents were used without further purification unless otherwise stated.

Nuclear magnetic resonance (NMR) Spectroscopy

NMR spectroscopy was measured on a ECZ400R (400 MHz) in the reported deuterated solvent CD_2Cl_2 and *d*^{8}-THF. All given chemical shifts in ^{1}H NMR spectra are calibrated on the resonance signals of $CDHCl_2$ contained in CD_2Cl_2 (δ = 5.32 ppm), and *d*^{7}-THF contained in *d*^{8}-THF (δ = 3.58, 1.72 ppm). The ^{13}C NMR spectra are calibrated on the respective resonance signal of CD_2Cl_2 (δ = 53.84 ppm) and *d*^{8}-THF (δ = 67.21, 25.31 ppm).[1] All other chemical shifts were referenced to Ξ values in IUPAC recommendations of 2008 using ^{2}H signal of the

deuterated solvent as internal reference.[2] The given multiplicities are phenomenological, thus the actual appearance of the signals is stated and not the theoretically expected one. The following abbreviations were used and analogously combined to designate multiplicities: s (singlet), d (doublet), t (triplet), q (quartet), m (multiplet). Evaluation of spectra was performed by using MESTRENOVA 14.0.0.[3] ^{103}Rh NMR spectra were measured with a triple resonance HPX probe as a gradient enhanced HMQC optimized for a ^{1}H, ^{103}Rh coupling constant of 5 Hz. To prevent nyquist fold-back for the indirectly measured dimension two spectra were measured. For first one a high sweep width (2ppt) was chosen and the second was measured on resonance and with a small sweepwidth (100 ppm) for the ^{103}Rh dimension.

X-Ray Diffraction (XRD)

X-Ray data was measured on a BRUKER X8 KAPPA APEX II diffractometer. The Data was collected at 100(2) K using graphite-monochromated Mo K_α radiation (λ_α = 0.71073 Å). The strategy for the data collection was evaluated by using the Smart software. The strategy for the data collection was gathered by the standard "ψ-ω scan techniques" and were scaled and reduced using Saint+software. The structures were solved by using Olex2[4] and XT[5] structure solution program using Intrinsic Phasing and refined with the XL refinement package[6] using at Least Squares minimization. Crystal drawings were generated with Mercury[7] and POV.Ray[8]. Crystallographic tables were generated with publcif.[9] Deposition numbers CCDC 2416649-2416650 contain the supplementary crystallographic data for this paper. This data is provided free of charge by the joint Cambridge Crystallographic Data Centre and Fachinformationszentrum Karlsruhe Access Structures service www.ccdc.cam.ac.uk/structures.

Quantum Chemical Calculations

All investigated structures were optimized at the r^{2}SCAN-3c level[10] using the ORCA program package, version 6.0.0.[11] This implies a scalar relativistic effective-core potential for Rh. All calculations included a continuum solvent model (CPCM with parametrization for CH_2Cl_2), a tight DFT grid (DefGrid3), and tight optimization criteria (VeryTightSCF and VeryTightOpt). All structures were verified to be proper minima by harmonic vibrational frequency analysis.

Nuclear shieldings and thus NMR chemical shifts were calculated using the AMS program package,[12] the PBE0 functional,[13] finer numerical settings (NumericalQuality VeryGood) and the TZ2P basis set (QZ4P-J at the Rh center) from the ADF library. It is known that ^{103}Rh shifts are well described by global hybrid functionals with moderate exact-exchange admixtures.[14] Relativistic effects were included at the two-component (SO)-ZORA level using a Gaussian model for the nuclear charge and magnetic moment. The shielding calculations included Autschbach's corrected exchange-correlation kernel.[15] EPR g-tensors were

calculated at the same level of theory using the PBE0 functional with a modified exact exchange admixture of 40 %. Energy decomposition analyses (EDA)[16] and extended transition-state analyses with natural orbitals for chemical valence (ETS-NOCV)[17] were performed at the BP86-D4/TZ2P level,[18] using a ZORA Hamiltonian to include scalar relativistic effects. Additionally, natural population analysis (NPA)[19] and quantum theory of atoms in molecules (QTAIM)[20] calculations were performed at the same level.

Fourier-Transform Infrared Spectroscopy (FT-IR)

FT-IR-spectroscopy was measured on a FT BRUKER ALPHA IR-spectrometer. The sample was directly measured by ATR (attenuated total reflection) technique. Characteristic absorptions are given in wavenumbers $\tilde{\nu}$ [cm^{-1}] and intensities are stated as vs (very strong), s (strong), m (medium) and w (weak).

High Resolution Mass Spectrometry (HRMS)

HRMS was performed using an AGILENT 6210 ESI-TOF spectrometer by electrospray ionization (ESI).

Elemental Analysis

Elemental analysis war performed by using a VARIO EL (Fa. ELEMENTAR) analyzer. Due to the nature of the highly fluorinated compounds, increased deviations between theoretical and experimental values can occur.

Electrochemistry

Cyclic voltammograms (CV) were recorded with an Interface 1010 B with a conventional three-electrode configuration of a platinum working electrode, a platinum auxiliary electrode and a platinum reference electrode. The measurement was carried out starting from 0 V to the reduction, followed by the oxidation. The measurement was carried out in anhydrous degassed THF containing 0.1 M [NBu_4][PF_6] (dried, 98% analyte grade) as supporting electrolyte. The software OriginPro 2017G was used to plot the data.[21]

EPR Spectroscopy

The EPR spectra were recorded in the X-band at −196 °C with a Magnetech MS 5000 spectrometer. The samples were sealed in an Argon atmosphere in 4 mm PFA tubes. Simulations were performed with Easyspin.[22]

Experimental Part

$[NEt_4][C_5(CF_3)_5]$

$[NEt_4][C_5(CF_3)_5]$ was prepared according to the improved published procedure of Sievers *et. al.*[46] **^{1}H NMR** (401 MHz, CD_2Cl_2, rt) δ [ppm] = 2.98 (q, $^3J_{H,H}$ = 7.3 Hz, 8H), 1.21 (t, $^3J_{H,H}$ = 7.3 Hz, $^4J_{H,H}$ = 1.8 Hz, 12H). **^{19}F NMR** (377 MHz, CD_2Cl_2, rt) δ [ppm] = −50.6 (s, 15F).

$[Rh(C_5Me_5)(C_5(CF_3)_5)][BF_4]$ $[1]^+[BF_4]^-$

In a dried 100 mL Schlenk tube $[Rh(C_5Me_5)Cl_2]_2$ (280 mg, 0.45 mmol, 1.0 equiv.) and $[NEt_4][C_5(CF_3)_5]$ (460 mg, 0.86 mmol, 1.9 equiv.) were dissolved in anhydrous CH_2Cl_2 (20 mL), upon which $[Ag][BF_4]$ (370 mg, 1.89 mmol, 4.2 equiv.) was added to the solution and the mixture was stirred for 48 h. The reaction mixture was filtered, diluted with CH_2Cl_2 (30 mL) and washed with water (50 mL). The aqueous phase was extracted with CH_2Cl_2 (2 × 50 mL) and the combined organic phases were washed with water (150 mL), dried over anhydrous Na_2SO_4 and the solvent was removed under reduced pressure. The remaining residue was washed with Et_2O (6 × 5 mL) and subsequently dried under high vacuum to afford $[Rh(C_5Me_5)(C_5(CF_3)_5)][BF_4]$ (310 mg, 0.42 mmol, 49%) as a colorless solid. **^{1}H NMR** (401 MHz, CD_2Cl_2, rt) δ [ppm] = 2.14 (s, 15H). **^{13}C{^{1}H} NMR** (101 MHz, CD_2Cl_2, rt) δ [ppm] = 119.8 (d, $^1J_{C,F}$ = 278.7 Hz), 113.5 (d, $^1J_{C,Rh}$ = 8.1 Hz), 10.7 (s). **^{13}C{^{19}F} NMR** (100 MHz, CD_2Cl_2, rt) δ [ppm] = 119.8 (s), 113.5 (s), 97.2 (d, $^1J_{C,Rh}$ = 6.0 Hz), 10.7 (q, $^1J_{C,H}$ = 131.4 Hz). **^{19}F NMR** (377 MHz, CD_2Cl_2, rt) δ [ppm] = −51.8 (s, 15F), −152.5 (s, 4F, $^{10}BF_4$), −152.6 (s, 4F, $^{11}BF_4$). **^{1}H,^{103}Rh HMQC** (400 MHz/12.76 MHz, CD_2Cl_2, rt) δ(^{1}H)/δ(^{103}Rh) [ppm] = 2.14/ −9308. **FT-IR** (ATR) ṽ [cm^{-1}] = 2916 (w), 2848 (w), 1478 (m), 1422 (m), 1381 (m), 1189 (vs), 1151 (vs), 1014 (s), 755 (w), 645 (s), 589 (w). **HRMS** (ESI-TOF, positive) m/z for $[Rh(C_5Me_5)(C_5(CF_3)_5)]$ calculated: 642.9989; measured: 642.9956. **EA** ($[Rh(C_5Me_5)(C_5(CF_3)_5)][BF_4]$) calculated: C: 32.91%, H: 2.07%; measured: C: 33.02%, H: 2.32%.

$[Rh(C_5Me_5)(C_5(CF_3)_5)]$ [1]

In a dried 10 mL Schlenk tube $[Co(C_5H_5)_2]$ (7.0 mg, 37 µmol, 0.90 equiv.) was dissolved in anhydrous degassed THF (1.0 mL) and cooled to −40 °C, upon which $[Rh(C_5Me_5)(C_5(CF_3)_5)][BF_4]$ (30 mg, 41 µmol, 1.0 equiv.) was added, generating a dark blue solution. The reaction mixture was stirred for 2 h, upon which an EPR spectra was obtained, confirming the generation of a radical species. Due to low solubility and decomposition at temperatures above −20 °C, it was not possible to obtain single crystals of $[Rh(C_5Me_5)(C_5(CF_3)_5)]$.

[Co(C_5Me_5)$_2$][Rh(C_5Me_5)($C_5(CF_3)_5$)] [Co(C_5Me_5)$_2$]$^+$[1]$^-$

In a dried 10 mL Schlenk tube [Rh(C_5Me_5)($C_5(CF_3)_5$)][BF_4] (30 mg, 41 µmol, 1.0 equiv.) was dissolved in degassed anhydrous THF (1.0 mL) and cooled to −75 °C. In a second 10 mL Schlenk tube [Co(C_5Me_5)$_2$] (40 mg, 0.12 mmol, 3.0 equiv.) was dissolved in degassed anhydrous THF (1.0 mL), which was added dropwise to the reaction mixture until a color change from a yellow to a blue and then further to a red solution occurred. The reaction mixture was stirred for 30 minutes, upon which low temperature NMR spectra at −80 °C was obtained and revealed a complete conversion to [Co(C_5Me_5)$_2$][Rh(C_5Me_5)($C_5(CF_3)_5$)] with only small traces of decomposition. Subsequently the reaction mixture was layered with degassed anhydrous *n*-hexane (6 mL) and the vessel was stored at −75 °C for 10 days, causing red crystals to form. **^{1}H NMR** (401 MHz, THF-d^8, −80 °C) δ [ppm] = 1.77 (s, 30H), 1.71 (s, 15H). **^{13}C{^{1}H} NMR** (101 MHz, THF-d^8, −80 °C) δ [ppm] = 95.2 (s), 92.3 (d, $^1J_{C,Rh}$ = 6.0 Hz), 11.4 (s), 8.0 (s). **^{19}F NMR** (377 MHz, THF-d^8, −80 °C) δ [ppm] = −49.3 (s, 3F), −53.3 (s, 6F), −58.1 (s, 6F). **^{1}H,^{103}Rh HMQC** (400 MHz/12.76 MHz, THF-d^8, −80 °C) δ(^{1}H)/δ(^{103}Rh) [ppm] = 1.71/−6895.

NMR Spectra

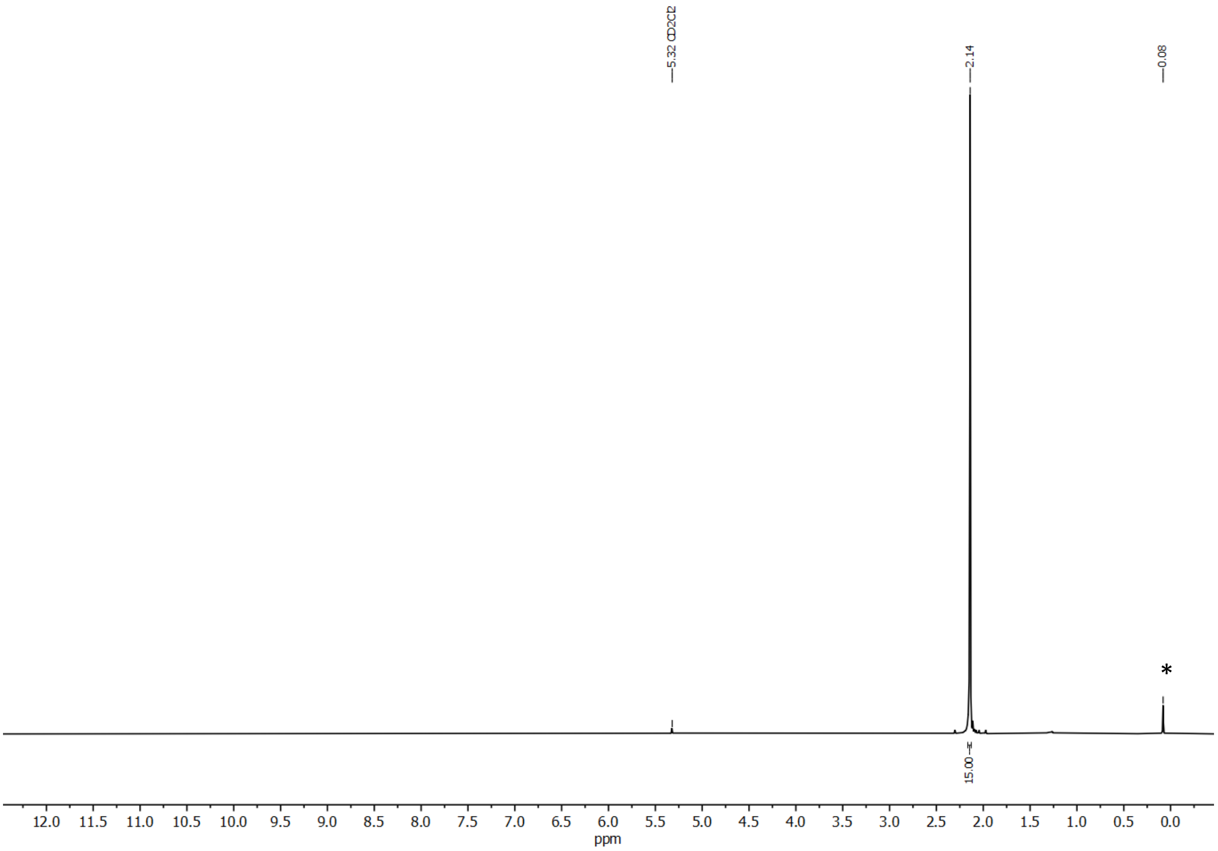

Figure S1. ^1H NMR (401 MHz, CD_2Cl_2, rt) spectrum of $[Rh(C_5Me_5)(C_5(CF_3)_5)][BF_4]$. *The impurity at 0.08 ppm contained in the deuterated solvent is probably silicon grease.

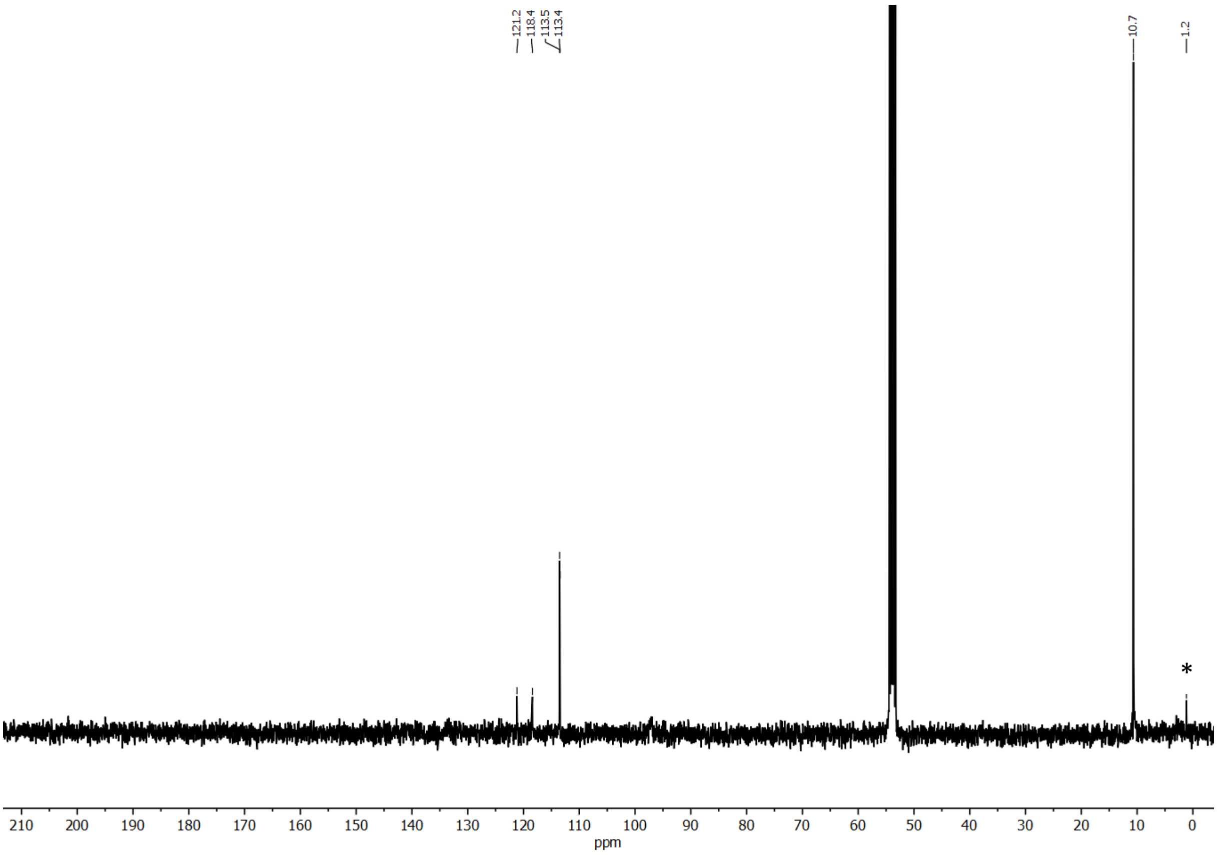

Figure S2. $^{13}C\{^{1}H\}$ NMR (101 MHz, CD_2Cl_2, rt) spectrum of $[Rh(C_5Me_5)(C_5(CF_3)_5)][BF_4]$. *The impurity at 1.2 ppm contained in the deuterated solvent is probably silicone grease.

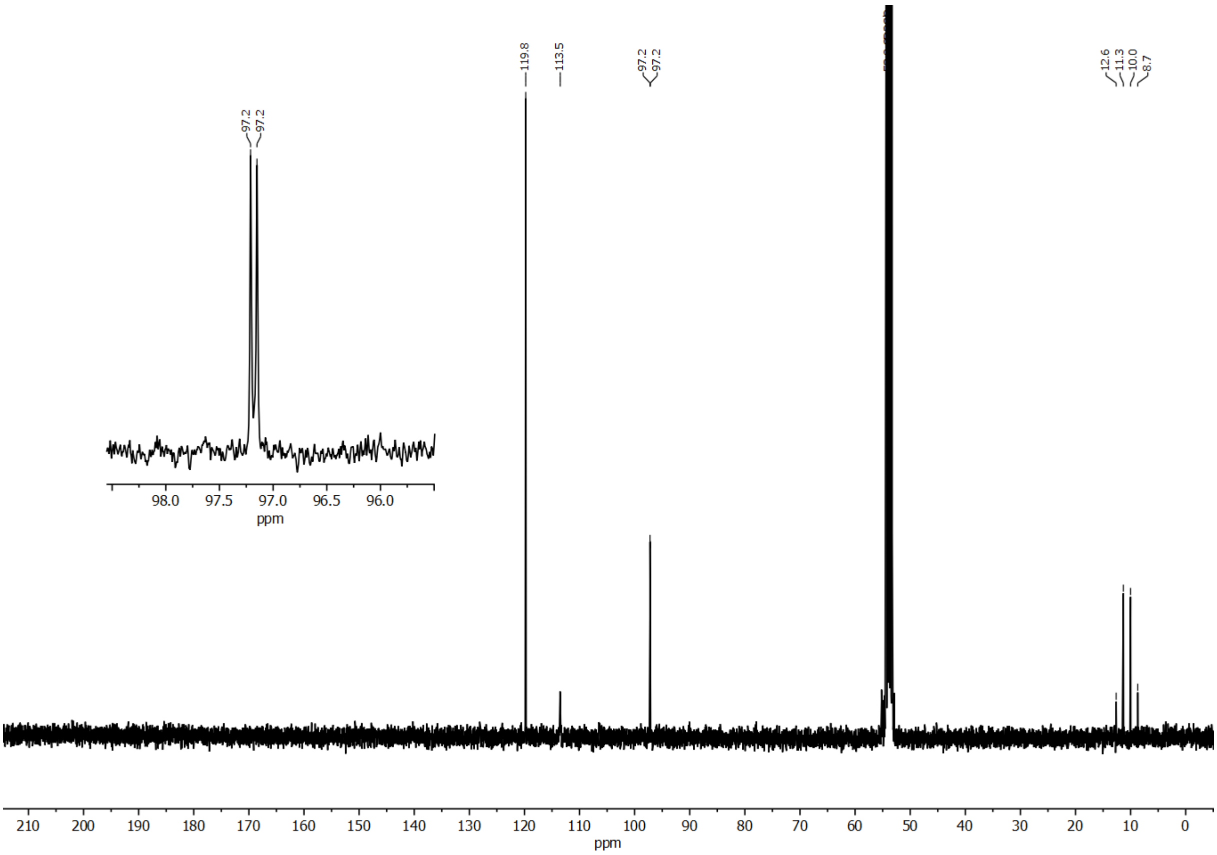

Figure S3. $^{13}C\{^{19}F\}$ NMR (100 MHz, CD_2Cl_2, rt) spectrum of $[Rh(C_5Me_5)(C_5(CF_3)_5)][BF_4]$.

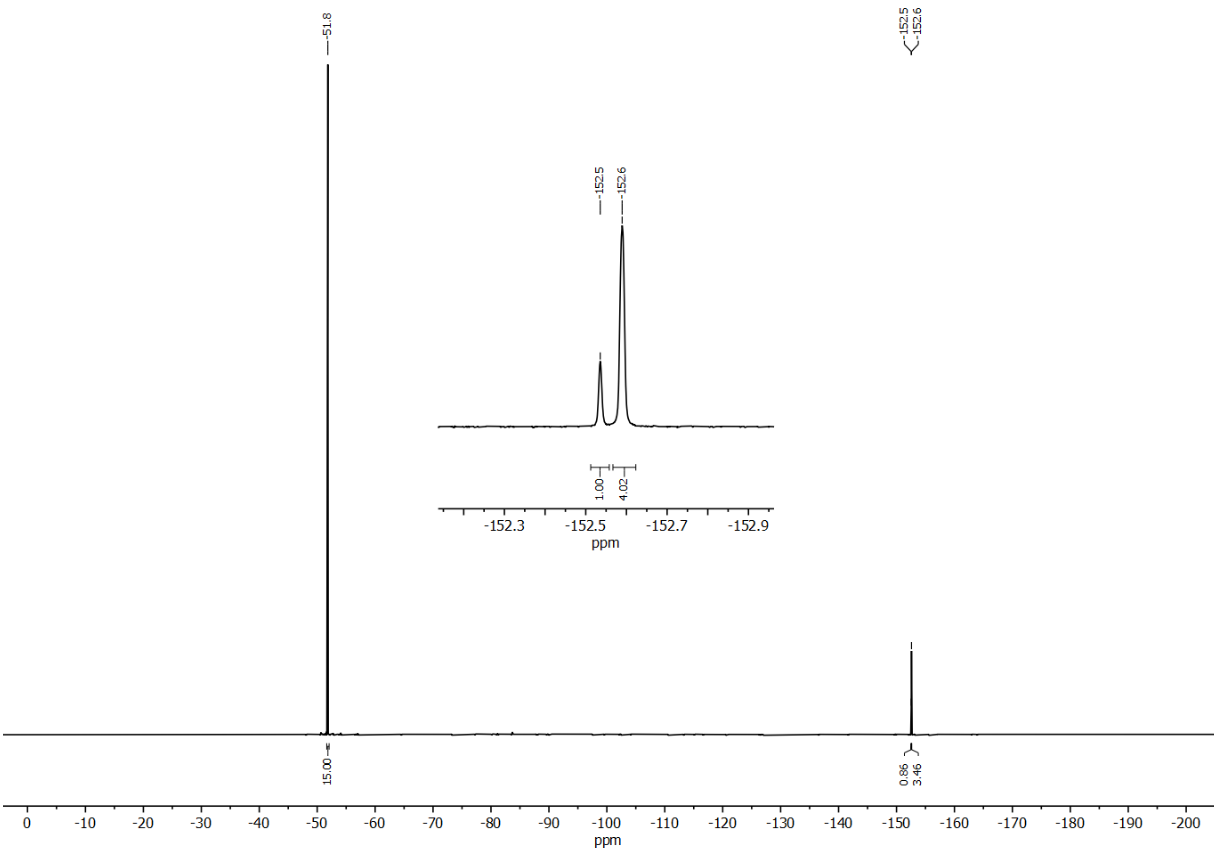

Figure S4. ^{19}F NMR (377 MHz, CD_2Cl_2, rt) spectrum of $[Rh(C_5Me_5)(C_5(CF_3)_5)][BF_4]$.

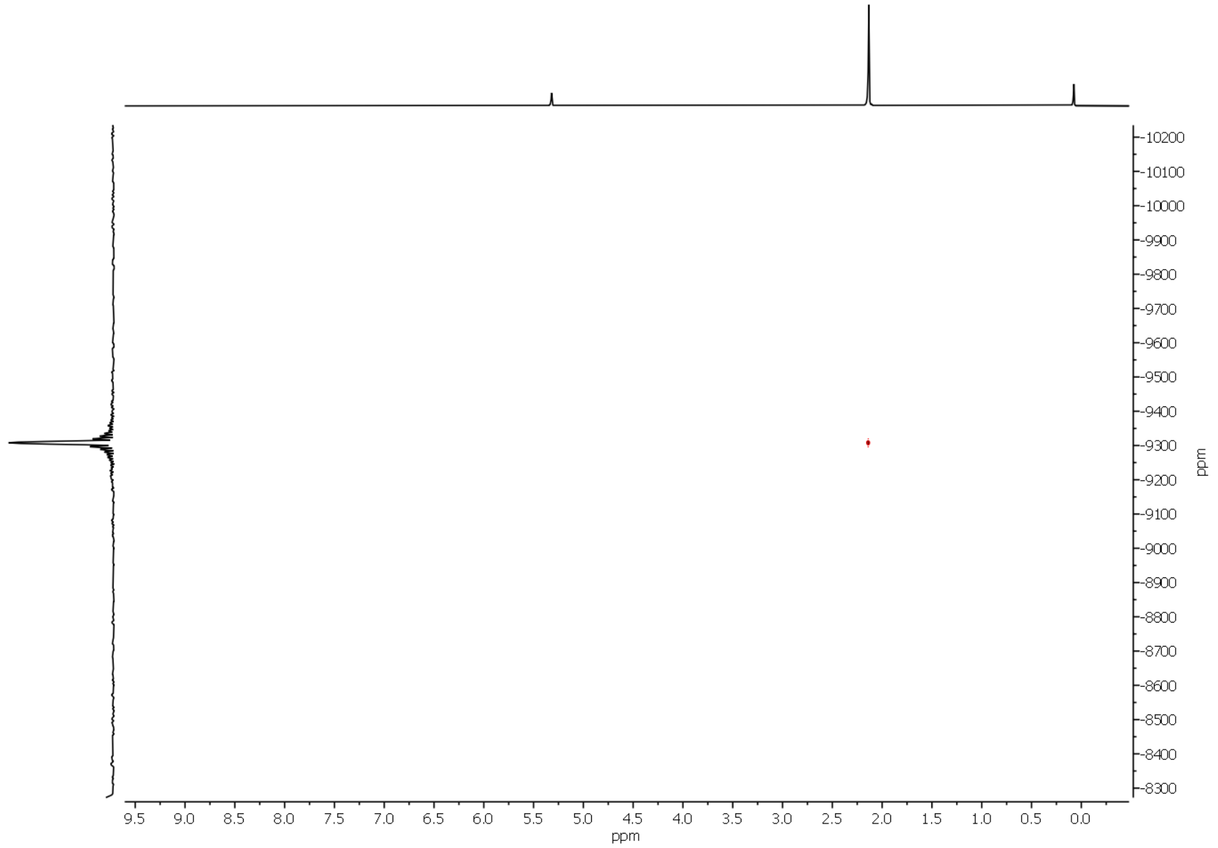

Figure S5. $^{1}H,^{103}Rh$ HMQC (400 MHz/12.76 MHz, CD_2Cl_2, rt) spectrum of $[Rh(C_5Me_5)(C_5(CF_3)_5)][BF_4]$. $\delta(^{1}H)/\delta(^{103}Rh)$ [ppm] = 2.14/ −9308.

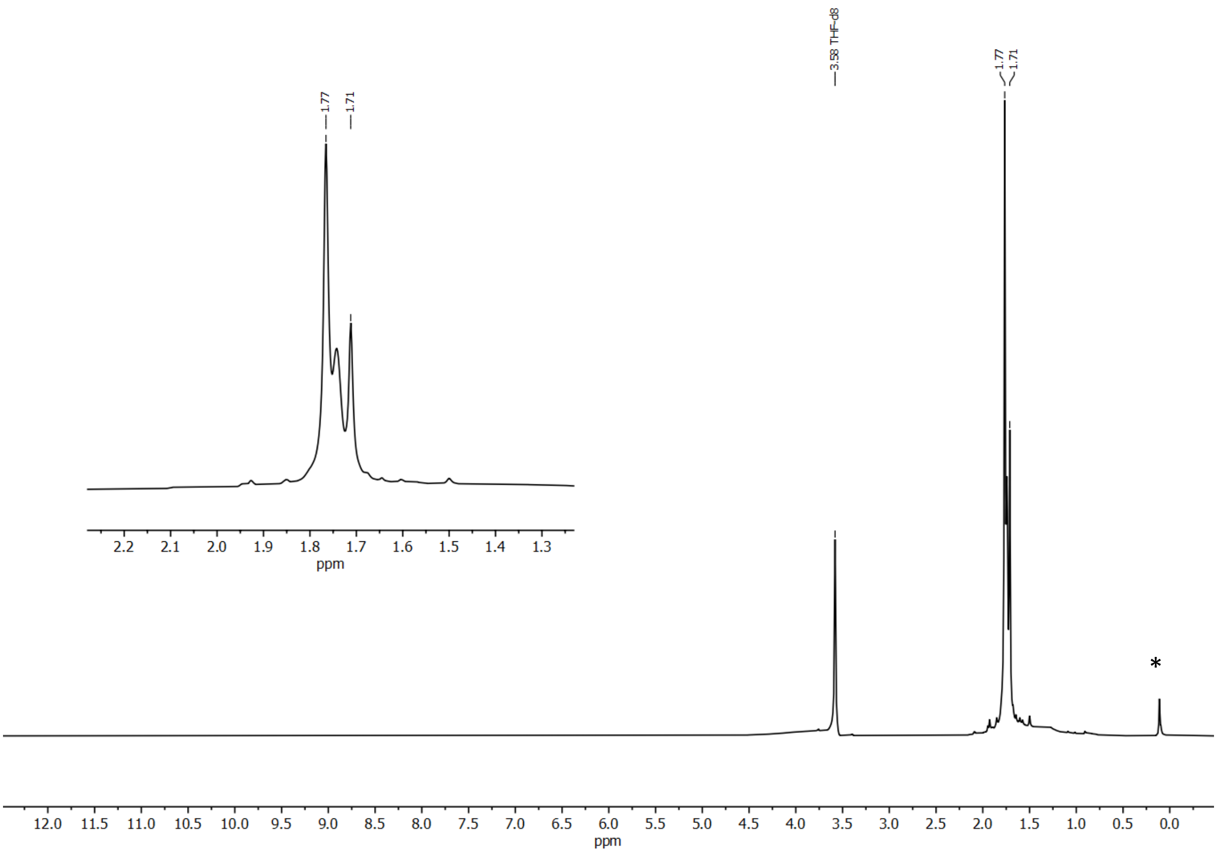

Figure S6. ^{1}H NMR (401 MHz, THF-d^{8}, −80 °C) spectrum of $[Co(C_5Me_5)_2][Rh(C_5Me_5)(C_5(CF_3)_5)]$, solvent residual signals at δ [ppm] = 1.74 and 3.58. *The Impurity at 0.11 ppm contained in the deuterated solvent is probably silicon grease.

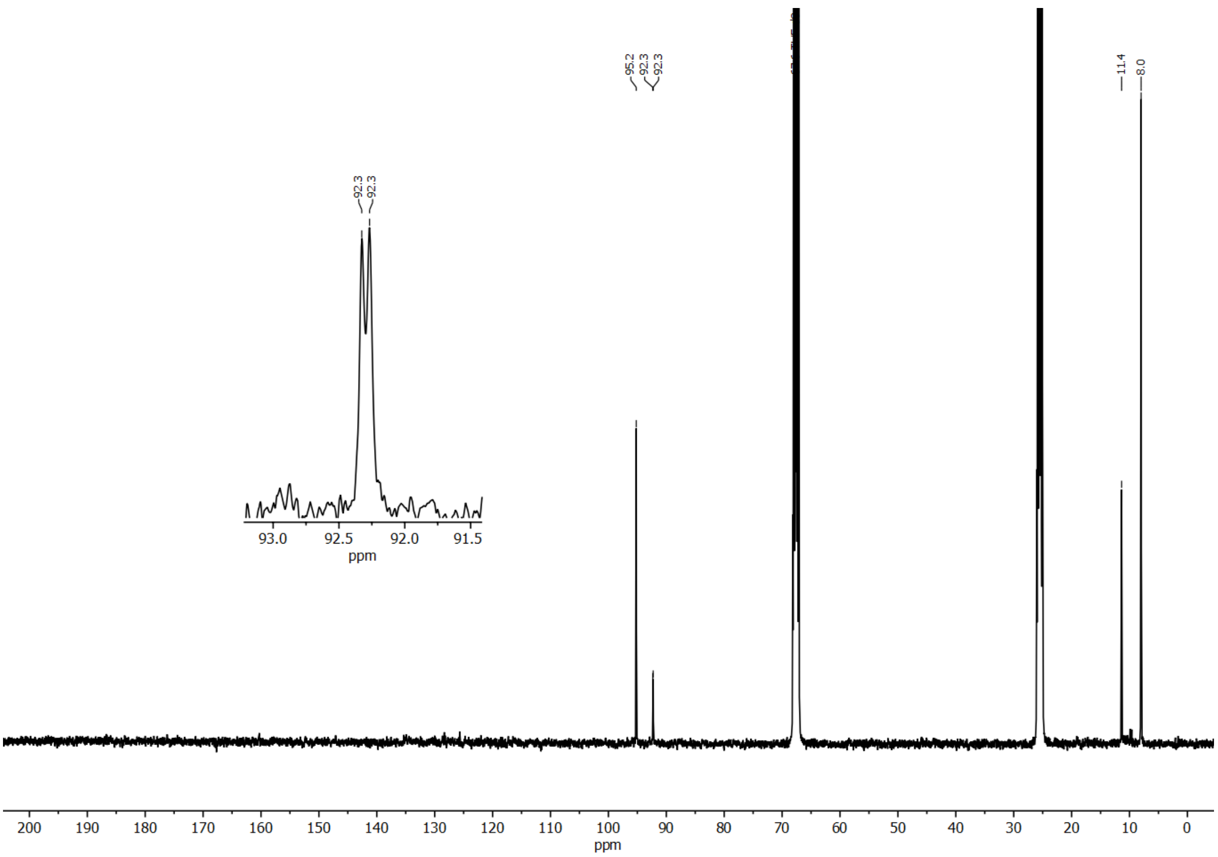

Figure S7. $^{13}C\{^{1}H\}$ NMR (101 MHz, THF-d^8, −80 °C) spectrum of $[Co(C_5Me_5)_2]$ $[Rh(C_5Me_5)(C_5(CF_3)_5)]$.

In our experience, the ^{13}C signals associated to the $[C_5(CF_3)_5]^-$ moiety are usually very challenging to resolve and typically only detected after long measurement times in $^{13}C\{^{19}F\}$ spectra.

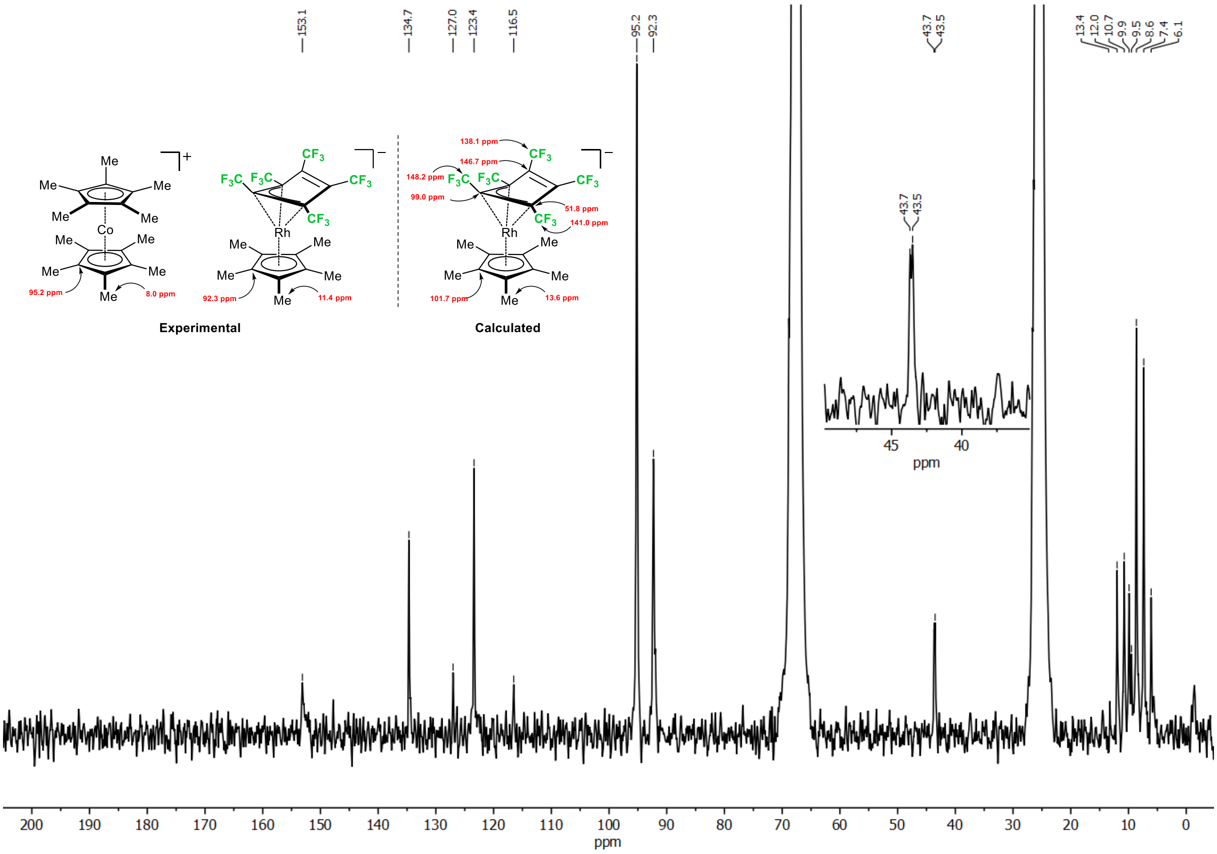

Figure S8. $^{13}C\{^{19}F\}$ NMR (101 MHz, THF-*d*[8], −80 °C) spectrum of the reaction mixture containing $[Co(C_5Me_5)_2][Rh(C_5Me_5)(C_5(CF_3)_5)]$ after a measurement time of 2 hours. Upon longer measurement times an increase of the free ligand $[C_5(CF_3)_5]^-$ was observed at −70 °C, which indicated a slow decomposition of the target compound. The solvent was slowly added to a precooled NMR tube (−80 °C) containing the starting materials, which was done 30 minutes before conducting NMR experiment. While the number of observed NMR signals correspond to the number of expected signals, the deviation of the calculated chemical shifts compared to the experimentally observed did not allow for the assignment of the individual carbon atoms of the η^3 bound $[C_5(CF_3)_5]^-$ ligand.

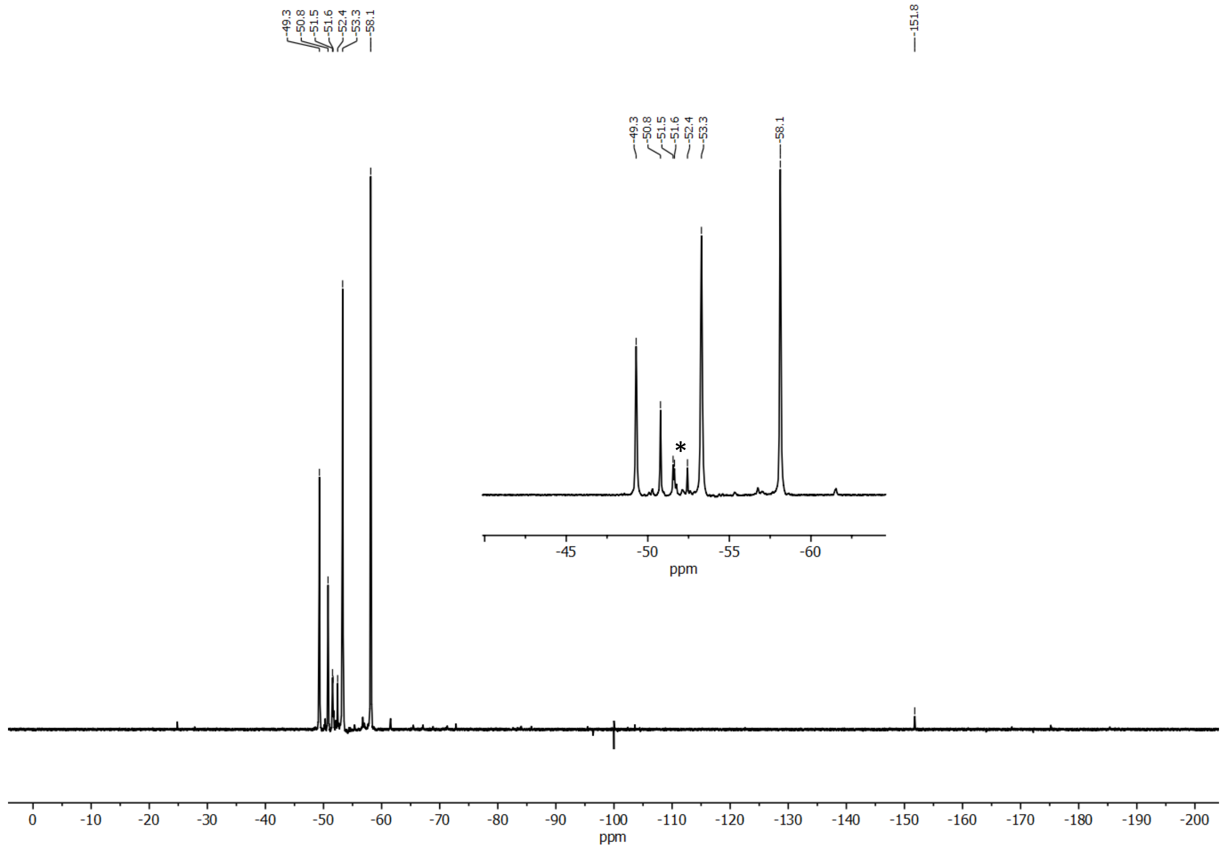

Figure S9. ^{19}F NMR (377 MHz, THF-*d*^{8}, −80 °C) spectrum of the reaction mixture containing $[Co(C_5Me_5)_2][Rh(C_5Me_5)(C_5(CF_3)_5)]$.

Comment: The signal at −50.8 ppm corresponds to uncoordinated $[C_5(CF_3)_5]^-$ ligand, while the signal at −151.8 ppm corresponds to the $[BF_4]^-$ signal of the byproduct $[Co(C_5Me_5)_2][BF_4]$. *Unknown decomposition products at −51.5 ppm, −51.6 ppm and −52.4 ppm. The peak at −100 ppm is an artefact (center of the spectrum).

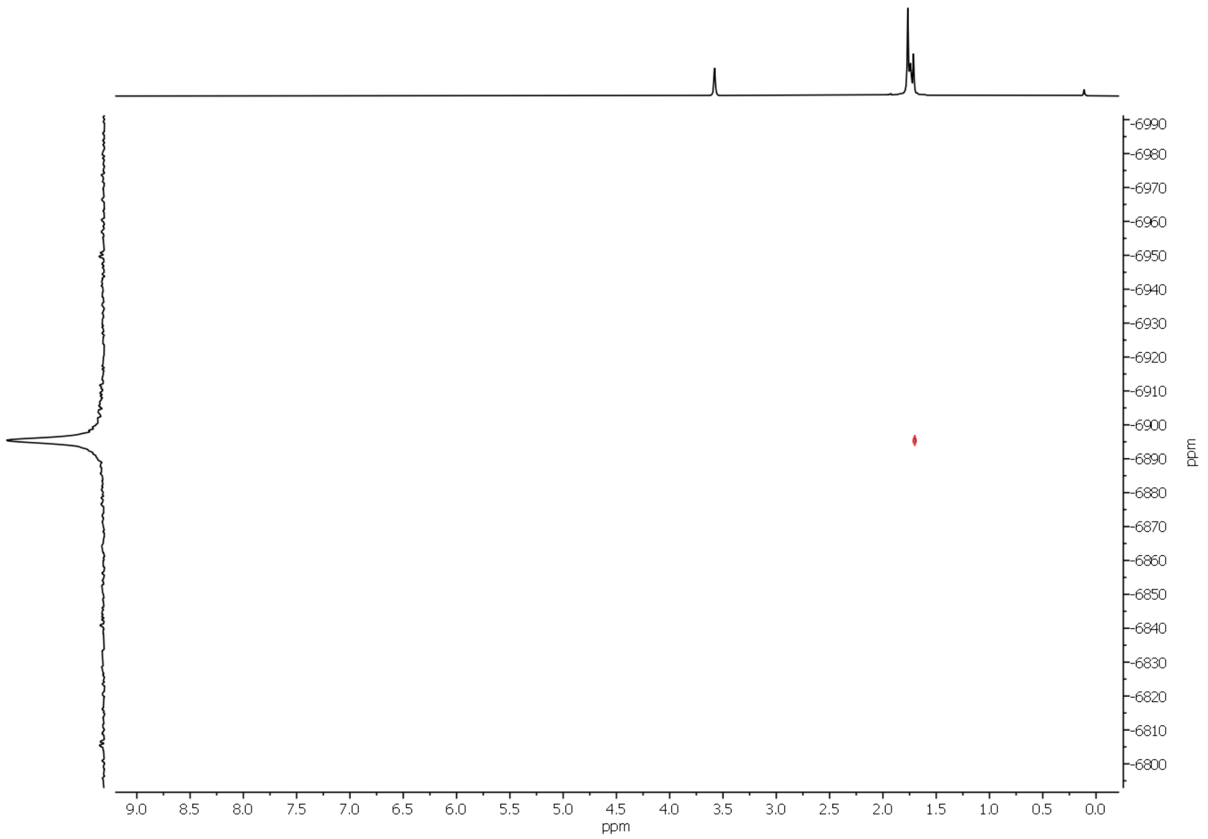

Figure S10. $^{1}H,^{103}Rh$ HMQC (400 MHz/12.76 MHz, THF-d^{8}, −80 °C) spectrum of $[Co(C_5Me_5)_2][Rh(C_5Me_5)(C_5(CF_3)_5)]$. $\delta(^{1}H)/\delta(^{103}Rh)$ [ppm] = 1.71/−6895.

Crystallographic Data

Table S1.Crystallographic Data of [Rh(C_5Me_5)($C_5(CF_3)_5$)][BF_4].

Identification code	CCDC 2416649
Empirical formula	$C_{20}H_{15}BF_{19}Rh$
Formula weight	730.04
Temperature/K	100.00
Crystal system	triclinic
Space group	$P\bar{1}$
a/Å	9.5995(7)
b/Å	15.7518(11)
c/Å	16.7157(11)
α/°	77.096(3)
β/°	88.756(2)
γ/°	79.582(3)
Volume/Å^3	2422.7(3)
Z	4
ρ_{calc}g/cm^3	2.002
μ/mm^{-1}	0.861
F(000)	1424.0
Crystal size/mm^3	0.1 × 0.1 × 0.1
Radiation	MoKα (λ = 0.71073)
2Θ range for data collection/°	4.066 to 50.838
Index ranges	-11 ≤ h ≤ 11, -18 ≤ k ≤ 18, -20 ≤ l ≤ 20
Reflections collected	40253
Independent reflections	8786 [R_{int} = 0.0963, R_{sigma} = 0.0757]
Data/restraints/parameters	8786/0/749
Goodness-of-fit on F^2	1.074
Final R indexes [I>=2σ (I)]	R_1 = 0.0761, wR_2 = 0.1365
Final R indexes [all data]	R_1 = 0.1159, wR_2 = 0.1535
Largest diff. peak/hole / e Å^{-3}	1.41/-1.37

Table S2. Crystallographic Data of $[Co(C_5Me_5)_2][Rh(C_5Me_5)(C_5(CF_3)_5)]$.

Identification code	CCDC 2416650
Empirical formula	$C_{40}H_{45}CoF_{15}Rh$
Formula weight	972.60
Temperature/K	100.0
Crystal system	monoclinic
Space group	$P2_1/n$
a/Å	15.8428(14)
b/Å	14.2130(10)
c/Å	17.7386(14)
α/°	90
β/°	105.622(3)
γ/°	90
Volume/Å^3	3846.7(5)
Z	4
ρ_{calc}g/cm^3	1.679
μ/mm^{-1}	0.963
F(000)	1968.0
Crystal size/mm^3	0.1 × 0.1 × 0.1
Radiation	MoKα (λ = 0.71073)
2Θ range for data collection/°	3.916 to 50.808
Index ranges	-19 ≤ h ≤ 19, -17 ≤ k ≤ 17, -21 ≤ l ≤ 21
Reflections collected	41777
Independent reflections	7055 [R_{int} = 0.1141, R_{sigma} = 0.0698]
Data/restraints/parameters	7055/0/529
Goodness-of-fit on F^2	1.082
Final R indexes [I>=2σ (I)]	R_1 = 0.0587, wR_2 = 0.1410
Final R indexes [all data]	R_1 = 0.0927, wR_2 = 0.1648
Largest diff. peak/hole / e Å^{-3}	1.14/-1.39

Table S3. Bond Lengths for $[Rh(C_5Me_5)(C_5(CF_3)_5)][BF_4]$.

Atom	Atom	Length/Å	Atom	Atom	Length/Å
Rh2	C23	2.224(9)	F14	C10	1.328(11)
Rh2	C32	2.175(8)	F27	C29	1.313(11)
Rh2	C22	2.222(9)	F11	C9	1.313(11)
Rh2	C34	2.155(8)	F2	C6	1.346(12)
Rh2	C25	2.215(8)	F28	C30	1.335(10)
Rh2	C24	2.224(7)	F34	B1	1.384(13)
Rh2	C31	2.202(9)	F30	C30	1.325(11)
Rh2	C35	2.165(9)	F18	C26	1.323(11)
Rh2	C33	2.172(8)	C4	C5	1.432(12)
Rh2	C21	2.223(8)	C4	C3	1.415(12)
Rh1	C4	2.229(8)	C4	C9	1.539(12)
Rh1	C5	2.213(8)	C5	C1	1.423(12)
Rh1	C13	2.202(8)	C5	C10	1.486(13)
Rh1	C12	2.179(8)	C13	C12	1.437(12)
Rh1	C1	2.201(8)	C13	C14	1.433(13)
Rh1	C14	2.181(8)	C13	C18	1.480(12)
Rh1	C2	2.231(9)	C23	C22	1.422(12)
Rh1	C3	2.211(8)	C23	C24	1.413(11)
Rh1	C15	2.175(9)	C23	C28	1.518(12)
Rh1	C11	2.178(8)	C12	C17	1.504(12)
F38	B2	1.380(11)	C12	C11	1.410(13)
F10	C9	1.353(11)	C32	C31	1.436(12)
F13	C10	1.320(10)	C32	C33	1.443(13)
F15	C10	1.354(11)	C32	C37	1.485(12)
F23	C28	1.340(11)	C22	C21	1.445(13)
F35	B2	1.386(12)	C22	C27	1.504(12)
F7	C8	1.316(10)	C1	C2	1.440(13)
F32	B1	1.394(11)	C1	C6	1.523(13)
F8	C8	1.339(12)	C14	C15	1.429(13)
F20	C27	1.334(11)	C14	C19	1.499(12)
F29	C30	1.326(11)	C34	C35	1.439(13)
F22	C28	1.324(11)	C34	C33	1.421(12)
F33	B1	1.380(12)	C34	C39	1.502(12)
F6	C7	1.345(11)	C25	C24	1.441(12)
F19	C27	1.320(11)	C25	C21	1.418(12)
F37	B2	1.382(13)	C25	C30	1.508(13)
F9	C8	1.339(11)	C24	C29	1.525(12)
F31	B1	1.371(13)	F21	C27	1.317(11)
F36	B2	1.401(12)	C31	C35	1.421(13)
F25	C29	1.357(10)	C31	C36	1.486(13)
F3	C6	1.338(12)	C2	C3	1.439(12)
F5	C7	1.327(13)	C2	C7	1.504(13)
F1	C6	1.314(11)	C35	C40	1.494(12)
F16	C26	1.340(12)	C33	C38	1.483(13)
F26	C29	1.302(11)	C21	C26	1.503(12)
F17	C26	1.315(11)	C3	C8	1.501(14)
F24	C28	1.313(11)	C15	C11	1.462(13)

Table S3. Bond Lengths for [Rh(C_5Me_5)($C_5(CF_3)_5$)][BF_4].

Atom	Atom	Length/Å	Atom	Atom	Length/Å
F12	C9	1.327(11)	C15	C20	1.496(13)
F4	C7	1.331(12)	C11	C16	1.501(13)

Table S4. Bond Angles for $[Rh(C_5Me_5)(C_5(CF_3)_5)][BF_4]$.

Atom	Atom	Atom	Angle/˚	Atom	Atom	Atom	Angle/˚
C23	Rh2	C24	37.0(3)	C15	C14	C13	110.1(8)
C32	Rh2	C23	137.7(3)	C15	C14	C19	124.4(9)
C32	Rh2	C22	115.4(3)	C19	C14	Rh1	127.3(6)
C32	Rh2	C25	147.2(3)	C35	C34	Rh2	70.9(5)
C32	Rh2	C24	174.0(3)	C35	C34	C39	125.1(8)
C32	Rh2	C31	38.3(3)	C33	C34	Rh2	71.5(5)
C32	Rh2	C21	119.8(3)	C33	C34	C35	108.8(8)
C22	Rh2	C23	37.3(3)	C33	C34	C39	125.8(8)
C22	Rh2	C24	62.2(3)	C39	C34	Rh2	128.1(6)
C22	Rh2	C21	37.9(3)	C24	C25	Rh2	71.4(4)
C34	Rh2	C23	119.1(3)	C24	C25	C30	127.0(8)
C34	Rh2	C32	64.3(3)	C21	C25	Rh2	71.7(4)
C34	Rh2	C22	147.7(3)	C21	C25	C24	107.0(7)
C34	Rh2	C25	136.1(3)	C21	C25	C30	126.0(8)
C34	Rh2	C24	114.4(3)	C30	C25	Rh2	122.6(6)
C34	Rh2	C31	64.2(3)	C23	C24	Rh2	71.5(5)
C34	Rh2	C35	38.9(3)	C23	C24	C25	108.8(7)
C34	Rh2	C33	38.3(3)	C23	C24	C29	127.2(8)
C34	Rh2	C21	172.7(3)	C25	C24	Rh2	70.7(4)
C25	Rh2	C23	63.0(3)	C25	C24	C29	123.8(8)
C25	Rh2	C22	63.1(3)	C29	C24	Rh2	127.7(6)
C25	Rh2	C24	37.9(3)	F13	C10	F15	105.7(7)
C25	Rh2	C21	37.3(3)	F13	C10	F14	107.2(8)
C31	Rh2	C23	174.4(3)	F13	C10	C5	113.4(7)
C31	Rh2	C22	137.6(3)	F15	C10	C5	111.2(7)
C31	Rh2	C25	118.3(3)	F14	C10	F15	105.0(7)
C31	Rh2	C24	147.3(3)	F14	C10	C5	113.7(8)
C31	Rh2	C21	114.7(3)	C32	C31	Rh2	69.9(5)
C35	Rh2	C23	147.4(3)	C32	C31	C36	126.6(9)
C35	Rh2	C32	64.2(3)	C35	C31	Rh2	69.6(5)
C35	Rh2	C22	173.3(3)	C35	C31	C32	107.7(8)
C35	Rh2	C25	113.2(3)	C35	C31	C36	125.7(9)
C35	Rh2	C24	118.9(3)	C36	C31	Rh2	126.5(6)
C35	Rh2	C31	38.0(3)	C1	C2	Rh1	69.9(5)
C35	Rh2	C33	64.8(3)	C1	C2	C7	126.3(9)
C35	Rh2	C21	135.7(3)	C3	C2	Rh1	70.3(5)
C33	Rh2	C23	114.7(3)	C3	C2	C1	107.7(8)
C33	Rh2	C32	38.8(3)	C3	C2	C7	125.3(9)
C33	Rh2	C22	119.5(3)	C7	C2	Rh1	132.7(6)
C33	Rh2	C25	173.2(3)	C34	C35	Rh2	70.2(5)
C33	Rh2	C24	136.5(3)	C34	C35	C40	125.6(9)
C33	Rh2	C31	64.6(3)	C31	C35	Rh2	72.4(5)
C33	Rh2	C21	148.6(3)	C31	C35	C34	108.0(8)
C21	Rh2	C23	62.6(3)	C31	C35	C40	126.1(9)
C21	Rh2	C24	62.2(3)	C40	C35	Rh2	127.8(6)
C4	Rh1	C2	62.1(3)	C32	C33	Rh2	70.7(5)
C5	Rh1	C4	37.6(3)	C32	C33	C38	126.0(8)

Table S4. Bond Angles for [Rh(C_5Me_5)($C_5(CF_3)_5$)][BF_4].

Atom	Atom	Atom	Angle/˚	Atom	Atom	Atom	Angle/˚
C5	Rh1	C2	62.9(3)	C34	C33	Rh2	70.2(5)
C13	Rh1	C4	120.2(3)	C34	C33	C32	107.0(8)
C13	Rh1	C5	148.6(3)	C34	C33	C38	126.8(9)
C13	Rh1	C2	136.2(3)	C38	C33	Rh2	128.0(6)
C13	Rh1	C3	114.2(3)	C22	C21	Rh2	71.0(5)
C12	Rh1	C4	149.0(3)	C22	C21	C26	124.7(8)
C12	Rh1	C5	172.3(3)	C25	C21	Rh2	71.1(5)
C12	Rh1	C13	38.3(3)	C25	C21	C22	108.4(7)
C12	Rh1	C1	136.0(3)	C25	C21	C26	126.6(8)
C12	Rh1	C14	63.5(3)	C26	C21	Rh2	128.3(6)
C12	Rh1	C2	114.8(3)	F23	C28	C23	111.6(8)
C12	Rh1	C3	120.0(3)	F22	C28	F23	107.2(8)
C1	Rh1	C4	62.6(3)	F22	C28	C23	111.2(7)
C1	Rh1	C5	37.6(3)	F24	C28	F23	106.1(8)
C1	Rh1	C13	172.9(3)	F24	C28	F22	108.0(8)
C1	Rh1	C2	37.9(3)	F24	C28	C23	112.5(8)
C1	Rh1	C3	63.6(3)	F25	C29	C24	108.4(7)
C14	Rh1	C4	115.5(3)	F26	C29	F25	107.4(8)
C14	Rh1	C5	119.8(3)	F26	C29	F27	109.4(8)
C14	Rh1	C13	38.2(3)	F26	C29	C24	114.3(7)
C14	Rh1	C1	148.1(4)	F27	C29	F25	105.2(7)
C14	Rh1	C2	173.0(4)	F27	C29	C24	111.5(8)
C14	Rh1	C3	136.2(4)	C4	C3	Rh1	72.1(5)
C3	Rh1	C4	37.2(3)	C4	C3	C2	107.5(8)
C3	Rh1	C5	63.3(3)	C4	C3	C8	126.7(8)
C3	Rh1	C2	37.8(3)	C2	C3	Rh1	71.9(5)
C15	Rh1	C4	135.9(3)	C2	C3	C8	125.8(8)
C15	Rh1	C5	113.2(3)	C8	C3	Rh1	120.0(6)
C15	Rh1	C13	64.8(3)	F20	C27	C22	113.0(8)
C15	Rh1	C12	64.5(3)	F19	C27	F20	107.0(8)
C15	Rh1	C1	118.4(4)	F19	C27	C22	112.2(8)
C15	Rh1	C14	38.3(4)	F21	C27	F20	103.9(8)
C15	Rh1	C2	148.2(4)	F21	C27	F19	108.1(9)
C15	Rh1	C3	172.3(3)	F21	C27	C22	112.1(8)
C15	Rh1	C11	39.2(3)	C14	C15	Rh1	71.1(5)
C11	Rh1	C4	173.0(4)	C14	C15	C11	106.1(8)
C11	Rh1	C5	135.8(4)	C14	C15	C20	125.6(9)
C11	Rh1	C13	64.2(3)	C11	C15	Rh1	70.5(5)
C11	Rh1	C12	37.8(3)	C11	C15	C20	127.9(9)
C11	Rh1	C1	113.7(3)	C20	C15	Rh1	128.7(6)
C11	Rh1	C14	64.0(3)	F29	C30	F28	107.1(8)
C11	Rh1	C2	119.3(3)	F29	C30	C25	112.5(7)
C11	Rh1	C3	148.1(4)	F28	C30	C25	112.9(8)
C5	C4	Rh1	70.6(4)	F30	C30	F29	103.6(8)
C5	C4	C9	126.9(8)	F30	C30	F28	109.1(8)
C3	C4	Rh1	70.7(4)	F30	C30	C25	111.1(8)
C3	C4	C5	109.2(7)	C12	C11	Rh1	71.2(5)

Table S4. Bond Angles for [Rh(C_5Me_5)($C_5(CF_3)_5$)][BF_4].

Atom	Atom	Atom	Angle/˚	Atom	Atom	Atom	Angle/˚
C3	C4	C9	123.8(8)	C12	C11	C15	107.9(8)
C9	C4	Rh1	129.0(6)	C12	C11	C16	126.3(9)
C4	C5	Rh1	71.8(5)	C15	C11	Rh1	70.3(5)
C4	C5	C10	124.9(8)	C15	C11	C16	125.5(9)
C1	C5	Rh1	70.7(5)	C16	C11	Rh1	128.1(6)
C1	C5	C4	107.5(8)	F10	C9	C4	110.5(8)
C1	C5	C10	127.3(8)	F12	C9	F10	107.4(8)
C10	C5	Rh1	127.9(6)	F12	C9	C4	111.9(7)
C12	C13	Rh1	70.0(5)	F11	C9	F10	105.8(8)
C12	C13	C18	127.0(8)	F11	C9	F12	109.0(8)
C14	C13	Rh1	70.1(5)	F11	C9	C4	112.0(8)
C14	C13	C12	106.0(8)	F6	C7	C2	112.7(8)
C14	C13	C18	126.8(8)	F5	C7	F6	107.2(9)
C18	C13	Rh1	127.3(6)	F5	C7	F4	106.3(8)
C22	C23	Rh2	71.3(5)	F5	C7	C2	112.4(9)
C22	C23	C28	126.1(8)	F4	C7	F6	106.7(8)
C24	C23	Rh2	71.5(5)	F4	C7	C2	111.1(9)
C24	C23	C22	108.2(8)	F16	C26	C21	112.5(8)
C24	C23	C28	125.7(8)	F17	C26	F16	105.3(8)
C28	C23	Rh2	124.3(6)	F17	C26	F18	108.5(9)
C13	C12	Rh1	71.7(4)	F17	C26	C21	113.9(7)
C13	C12	C17	124.4(8)	F18	C26	F16	103.1(8)
C17	C12	Rh1	127.1(6)	F18	C26	C21	112.7(8)
C11	C12	Rh1	71.1(5)	F3	C6	F2	104.3(8)
C11	C12	C13	109.8(8)	F3	C6	C1	112.0(8)
C11	C12	C17	125.7(8)	F1	C6	F3	108.4(9)
C31	C32	Rh2	71.8(5)	F1	C6	F2	108.4(8)
C31	C32	C33	108.5(8)	F1	C6	C1	112.7(8)
C31	C32	C37	126.7(9)	F2	C6	C1	110.7(9)
C33	C32	Rh2	70.5(5)	F7	C8	F8	107.8(8)
C33	C32	C37	124.7(8)	F7	C8	F9	107.7(8)
C37	C32	Rh2	125.5(6)	F7	C8	C3	113.4(8)
C23	C22	Rh2	71.4(5)	F8	C8	F9	105.0(8)
C23	C22	C21	107.5(7)	F8	C8	C3	110.8(8)
C23	C22	C27	126.5(8)	F9	C8	C3	111.8(8)
C21	C22	Rh2	71.1(5)	F38	B2	F35	110.9(8)
C21	C22	C27	125.9(8)	F38	B2	F37	110.6(9)
C27	C22	Rh2	125.4(6)	F38	B2	F36	109.1(8)
C5	C1	Rh1	71.7(5)	F35	B2	F36	108.6(8)
C5	C1	C2	108.1(8)	F37	B2	F35	109.5(8)
C5	C1	C6	126.2(9)	F37	B2	F36	108.1(8)
C2	C1	Rh1	72.2(5)	F33	B1	F32	108.0(8)
C2	C1	C6	125.7(8)	F33	B1	F34	108.1(9)
C6	C1	Rh1	121.3(6)	F31	B1	F32	109.9(8)
C13	C14	Rh1	71.7(5)	F31	B1	F33	111.7(8)
C13	C14	C19	125.4(9)	F31	B1	F34	109.5(9)
C15	C14	Rh1	70.6(5)	F34	B1	F32	109.6(8)

Table S5. Torsion Angles for [Rh(C_5Me_5)($C_5(CF_3)_5$)][BF_4].

A	B	C	D	Angle/˚	A	B	C	D	Angle/˚
Rh2	C23	C22	C21	62.3(6)	C1	C2	C3	C4	-3.6(9)
Rh2	C23	C22	C27	-120.8(9)	C1	C2	C3	C8	174.5(8)
Rh2	C23	C24	C25	-61.2(6)	C1	C2	C7	F6	-80.1(12)
Rh2	C23	C24	C29	123.7(8)	C1	C2	C7	F5	158.6(8)
Rh2	C23	C28	F23	-38.9(11)	C1	C2	C7	F4	39.5(13)
Rh2	C23	C28	F22	-158.5(6)	C14	C13	C12	Rh1	61.2(5)
Rh2	C23	C28	F24	80.2(9)	C14	C13	C12	C17	-175.9(7)
Rh2	C32	C31	C35	-59.6(6)	C14	C13	C12	C11	0.0(9)
Rh2	C32	C31	C36	121.2(9)	C14	C15	C11	Rh1	-62.6(6)
Rh2	C32	C33	C34	61.1(5)	C14	C15	C11	C12	-1.0(9)
Rh2	C32	C33	C38	-123.6(9)	C14	C15	C11	C16	174.1(8)
Rh2	C22	C21	C25	61.5(6)	C25	C24	C29	F25	-49.9(11)
Rh2	C22	C21	C26	-124.0(8)	C25	C24	C29	F26	69.8(11)
Rh2	C22	C27	F20	-32.1(12)	C25	C24	C29	F27	-165.4(8)
Rh2	C22	C27	F19	-153.2(7)	C25	C21	C26	F16	122.5(10)
Rh2	C22	C27	F21	84.9(10)	C25	C21	C26	F17	-117.8(10)
Rh2	C34	C35	C31	62.9(6)	C25	C21	C26	F18	6.5(13)
Rh2	C34	C35	C40	-122.8(9)	C24	C23	C22	Rh2	-62.3(6)
Rh2	C34	C33	C32	-61.5(6)	C24	C23	C22	C21	0.0(9)
Rh2	C34	C33	C38	123.2(9)	C24	C23	C22	C27	176.9(8)
Rh2	C25	C24	C23	61.7(6)	C24	C23	C28	F23	52.1(12)
Rh2	C25	C24	C29	-123.0(8)	C24	C23	C28	F22	-67.5(11)
Rh2	C25	C21	C22	-61.5(6)	C24	C23	C28	F24	171.2(8)
Rh2	C25	C21	C26	124.1(9)	C24	C25	C21	Rh2	63.1(6)
Rh2	C25	C30	F29	-43.2(11)	C24	C25	C21	C22	1.6(9)
Rh2	C25	C30	F28	-164.6(7)	C24	C25	C21	C26	-172.8(8)
Rh2	C25	C30	F30	72.5(9)	C24	C25	C30	F29	-133.6(9)
Rh2	C24	C29	F25	-140.8(7)	C24	C25	C30	F28	105.0(11)
Rh2	C24	C29	F26	-21.0(12)	C24	C25	C30	F30	-17.9(13)
Rh2	C24	C29	F27	103.8(9)	C10	C5	C1	Rh1	-123.4(8)
Rh2	C31	C35	C34	-61.5(6)	C10	C5	C1	C2	173.1(8)
Rh2	C31	C35	C40	124.3(9)	C10	C5	C1	C6	-7.6(14)
Rh2	C21	C26	F16	-143.4(7)	C31	C32	C33	Rh2	-62.2(6)
Rh2	C21	C26	F17	-23.7(13)	C31	C32	C33	C34	-1.0(9)
Rh2	C21	C26	F18	100.5(9)	C31	C32	C33	C38	174.3(8)
Rh1	C4	C5	C1	-62.1(6)	C2	C1	C6	F3	127.4(9)
Rh1	C4	C5	C10	124.0(8)	C2	C1	C6	F1	-110.0(10)
Rh1	C4	C3	C2	63.6(6)	C2	C1	C6	F2	11.5(12)
Rh1	C4	C3	C8	-114.5(8)	C2	C3	C8	F7	84.1(11)
Rh1	C4	C9	F10	130.4(7)	C2	C3	C8	F8	-154.6(8)
Rh1	C4	C9	F12	10.8(12)	C2	C3	C8	F9	-37.9(12)
Rh1	C4	C9	F11	-112.0(9)	C35	C34	C33	Rh2	61.4(6)
Rh1	C5	C1	C2	-63.4(6)	C35	C34	C33	C32	0.0(9)
Rh1	C5	C1	C6	115.8(8)	C35	C34	C33	C38	-175.3(8)
Rh1	C5	C10	F13	159.6(6)	C33	C32	C31	Rh2	61.3(6)
Rh1	C5	C10	F15	40.6(11)	C33	C32	C31	C35	1.7(9)

Table S5. Torsion Angles for [Rh(C_5Me_5)($C_5(CF_3)_5$)][BF_4].

A	B	C	D	Angle/˚	A	B	C	D	Angle/˚
Rh1	C5	C10	F14	-77.6(10)	C33	C32	C31	C36	-177.5(8)
Rh1	C13	C12	C17	122.9(8)	C33	C34	C35	Rh2	-61.8(6)
Rh1	C13	C12	C11	-61.2(6)	C33	C34	C35	C31	1.1(9)
Rh1	C13	C14	C15	60.5(6)	C33	C34	C35	C40	175.4(8)
Rh1	C13	C14	C19	-123.3(9)	C21	C22	C27	F20	-123.5(9)
Rh1	C12	C11	C15	-61.0(6)	C21	C22	C27	F19	115.4(10)
Rh1	C12	C11	C16	123.9(9)	C21	C22	C27	F21	-6.4(13)
Rh1	C1	C2	C3	-60.5(6)	C21	C25	C24	Rh2	-63.3(6)
Rh1	C1	C2	C7	128.8(9)	C21	C25	C24	C23	-1.6(9)
Rh1	C1	C6	F3	37.6(11)	C21	C25	C24	C29	173.7(7)
Rh1	C1	C6	F1	160.2(7)	C21	C25	C30	F29	47.0(12)
Rh1	C1	C6	F2	-78.3(10)	C21	C25	C30	F28	-74.4(12)
Rh1	C14	C15	C11	62.2(6)	C21	C25	C30	F30	162.7(8)
Rh1	C14	C15	C20	-124.5(9)	C17	C12	C11	Rh1	-122.5(8)
Rh1	C2	C3	C4	-63.8(6)	C17	C12	C11	C15	176.5(8)
Rh1	C2	C3	C8	114.3(8)	C17	C12	C11	C16	1.4(14)
Rh1	C2	C7	F6	15.5(15)	C28	C23	C22	Rh2	119.3(8)
Rh1	C2	C7	F5	-105.8(10)	C28	C23	C22	C21	-178.4(8)
Rh1	C2	C7	F4	135.2(8)	C28	C23	C22	C27	-1.5(14)
Rh1	C3	C8	F7	172.6(6)	C28	C23	C24	Rh2	-119.4(8)
Rh1	C3	C8	F8	-66.1(9)	C28	C23	C24	C25	179.4(8)
Rh1	C3	C8	F9	50.6(10)	C28	C23	C24	C29	4.3(14)
Rh1	C15	C11	C12	61.5(6)	C3	C4	C5	Rh1	60.5(6)
Rh1	C15	C11	C16	-123.3(9)	C3	C4	C5	C1	-1.7(9)
C4	C5	C1	Rh1	62.8(6)	C3	C4	C5	C10	-175.6(8)
C4	C5	C1	C2	-0.6(9)	C3	C4	C9	F10	38.6(12)
C4	C5	C1	C6	178.6(8)	C3	C4	C9	F12	-81.0(11)
C4	C5	C10	F13	66.1(11)	C3	C4	C9	F11	156.3(8)
C4	C5	C10	F15	-52.8(11)	C3	C2	C7	F6	110.7(11)
C4	C5	C10	F14	-171.1(8)	C3	C2	C7	F5	-10.5(13)
C4	C3	C8	F7	-98.2(11)	C3	C2	C7	F4	-129.6(10)
C4	C3	C8	F8	23.2(12)	C27	C22	C21	Rh2	120.6(9)
C4	C3	C8	F9	139.9(9)	C27	C22	C21	C25	-177.9(8)
C5	C4	C3	Rh1	-60.4(6)	C27	C22	C21	C26	-3.4(13)
C5	C4	C3	C2	3.3(9)	C30	C25	C24	Rh2	117.2(9)
C5	C4	C3	C8	-174.8(8)	C30	C25	C24	C23	178.9(8)
C5	C4	C9	F10	-135.5(9)	C30	C25	C24	C29	-5.8(13)
C5	C4	C9	F12	105.0(10)	C30	C25	C21	Rh2	-117.4(9)
C5	C4	C9	F11	-17.8(13)	C30	C25	C21	C22	-178.9(8)
C5	C1	C2	Rh1	63.1(6)	C30	C25	C21	C26	6.7(14)
C5	C1	C2	C3	2.6(9)	C9	C4	C5	Rh1	-124.8(8)
C5	C1	C2	C7	-168.1(8)	C9	C4	C5	C1	173.1(8)
C5	C1	C6	F3	-51.7(12)	C9	C4	C5	C10	-0.8(13)
C5	C1	C6	F1	70.9(12)	C9	C4	C3	Rh1	124.7(8)
C5	C1	C6	F2	-167.6(8)	C9	C4	C3	C2	-171.7(8)
C13	C12	C11	Rh1	61.6(6)	C9	C4	C3	C8	10.2(13)
C13	C12	C11	C15	0.6(9)	C7	C2	C3	Rh1	-129.0(9)

Table S5. Torsion Angles for $[Rh(C_5Me_5)(C_5(CF_3)_5)][BF_4]$.

A	B	C	D	Angle/˚	A	B	C	D	Angle/˚
C13	C12	C11	C16	-174.5(8)	C7	C2	C3	C4	167.2(8)
C13	C14	C15	Rh1	-61.1(6)	C7	C2	C3	C8	-14.7(14)
C13	C14	C15	C11	1.0(9)	C6	C1	C2	Rh1	-116.1(8)
C13	C14	C15	C20	174.3(8)	C6	C1	C2	C3	-176.6(8)
C23	C22	C21	Rh2	-62.5(6)	C6	C1	C2	C7	12.7(14)
C23	C22	C21	C25	-1.0(9)	C20	C15	C11	Rh1	124.4(9)
C23	C22	C21	C26	173.5(8)	C20	C15	C11	C12	-174.1(8)
C23	C22	C27	F20	60.2(12)	C20	C15	C11	C16	1.1(14)
C23	C22	C27	F19	-60.9(12)	C19	C14	C15	Rh1	122.6(8)
C23	C22	C27	F21	177.2(9)	C19	C14	C15	C11	-175.3(8)
C23	C24	C29	F25	124.5(9)	C19	C14	C15	C20	-2.0(14)
C23	C24	C29	F26	-115.8(10)	C39	C34	C35	Rh2	123.7(8)
C23	C24	C29	F27	9.0(12)	C39	C34	C35	C31	-173.4(8)
C12	C13	C14	Rh1	-61.1(5)	C39	C34	C35	C40	0.8(14)
C12	C13	C14	C15	-0.7(9)	C39	C34	C33	Rh2	-124.1(9)
C12	C13	C14	C19	175.6(8)	C39	C34	C33	C32	174.4(8)
C32	C31	C35	Rh2	59.7(6)	C39	C34	C33	C38	-0.8(14)
C32	C31	C35	C34	-1.8(9)	C18	C13	C12	Rh1	-122.2(8)
C32	C31	C35	C40	-175.9(8)	C18	C13	C12	C17	0.6(13)
C22	C23	C24	Rh2	62.2(6)	C18	C13	C12	C11	176.6(8)
C22	C23	C24	C25	1.0(9)	C18	C13	C14	Rh1	122.3(8)
C22	C23	C24	C29	-174.1(8)	C18	C13	C14	C15	-177.2(8)
C22	C23	C28	F23	-129.9(9)	C18	C13	C14	C19	-1.0(14)
C22	C23	C28	F22	110.6(10)	C37	C32	C31	Rh2	-121.2(9)
C22	C23	C28	F24	-10.8(13)	C37	C32	C31	C35	179.2(8)
C22	C21	C26	F16	-51.0(11)	C37	C32	C31	C36	0.0(14)
C22	C21	C26	F17	68.7(12)	C37	C32	C33	Rh2	120.3(8)
C22	C21	C26	F18	-167.0(8)	C37	C32	C33	C34	-178.6(8)
C1	C5	C10	F13	-106.6(10)	C37	C32	C33	C38	-3.3(14)
C1	C5	C10	F15	134.5(9)	C36	C31	C35	Rh2	-121.0(8)
C1	C5	C10	F14	16.2(12)	C36	C31	C35	C34	177.5(8)
C1	C2	C3	Rh1	60.2(6)	C36	C31	C35	C40	3.3(14)

Table S6. Bond Lengths for $[Co(C_5Me_5)_2][Rh(C_5Me_5)(C_5(CF_3)_5)]$.

Atom	Atom	Length/Å	Atom	Atom	Length/Å
Rh1	C1	2.007(6)	C33	C34	1.430(9)
Rh1	C5	2.216(7)	C33	C38	1.495(9)
Rh1	C2	2.179(7)	C32	C37	1.486(9)
Rh1	C15	2.189(7)	C35	C34	1.418(10)
Rh1	C13	2.214(6)	C35	C40	1.507(10)
Rh1	C14	2.227(7)	C21	C25	1.433(9)
Rh1	C12	2.214(6)	C21	C22	1.431(9)
Rh1	C11	2.271(7)	C21	C26	1.501(9)
Co1	C31	2.043(6)	C25	C24	1.436(9)
Co1	C33	2.040(6)	C25	C30	1.485(9)
Co1	C32	2.054(6)	C3	C2	1.494(9)
Co1	C35	2.046(7)	C3	C4	1.354(9)
Co1	C21	2.046(6)	C3	C8	1.481(9)
Co1	C25	2.048(7)	C34	C39	1.496(9)
Co1	C34	2.045(7)	C1	C6	1.477(9)
Co1	C22	2.055(7)	C1	C5	1.477(8)
Co1	C24	2.046(6)	C1	C2	1.482(8)
Co1	C23	2.045(7)	C22	C23	1.445(9)
F6	C7	1.350(7)	C22	C27	1.491(9)
F14	C10	1.340(8)	C5	C4	1.464(9)
F4	C7	1.354(8)	C5	C10	1.491(9)
F5	C7	1.358(7)	C24	C23	1.417(9)
F13	C10	1.360(8)	C24	C29	1.492(9)
F3	C6	1.345(7)	C2	C7	1.475(8)
F15	C10	1.351(8)	C15	C14	1.469(9)
F8	C8	1.361(8)	C15	C20	1.495(9)
F2	C6	1.331(8)	C15	C11	1.426(10)
F1	C6	1.363(8)	C13	C14	1.388(9)
F10	C9	1.360(9)	C13	C12	1.471(9)
F9	C8	1.341(8)	C13	C18	1.500(9)
F12	C9	1.327(8)	C4	C9	1.494(9)
F11	C9	1.349(8)	C14	C19	1.506(9)
F7	C8	1.329(9)	C23	C28	1.494(9)
C31	C32	1.439(9)	C12	C11	1.428(9)
C31	C35	1.412(10)	C12	C17	1.490(9)
C31	C36	1.504(9)	C16	C11	1.510(10)
C33	C32	1.420(9)			

Table S7. Bond Angles for $[Co(C_5Me_5)_2][Rh(C_5Me_5)(C_5(CF_3)_5)]$.

Atom	Atom	Atom	Angle/˚	Atom	Atom	Atom	Angle/˚
C1	Rh1	C5	40.6(2)	C4	C3	C2	107.8(5)
C1	Rh1	C2	41.2(2)	C4	C3	C8	126.6(6)
C1	Rh1	C15	140.3(3)	C8	C3	C2	124.2(6)
C1	Rh1	C13	156.3(3)	C33	C34	Co1	69.3(4)
C1	Rh1	C14	159.0(2)	C33	C34	C39	126.7(6)
C1	Rh1	C12	138.1(2)	C35	C34	Co1	69.8(4)
C1	Rh1	C11	131.7(2)	C35	C34	C33	107.6(6)
C5	Rh1	C14	118.5(2)	C35	C34	C39	125.6(6)
C5	Rh1	C11	142.2(2)	C39	C34	Co1	128.1(5)
C2	Rh1	C5	62.1(2)	C6	C1	Rh1	135.4(5)
C2	Rh1	C15	177.2(2)	C6	C1	C2	126.4(6)
C2	Rh1	C13	115.3(2)	C5	C1	Rh1	77.3(4)
C2	Rh1	C14	138.6(2)	C5	C1	C6	125.6(5)
C2	Rh1	C12	117.7(2)	C5	C1	C2	99.9(5)
C2	Rh1	C11	144.8(3)	C2	C1	Rh1	75.6(4)
C15	Rh1	C5	117.4(2)	C21	C22	Co1	69.2(4)
C15	Rh1	C13	63.3(2)	C21	C22	C23	107.3(6)
C15	Rh1	C14	38.8(2)	C21	C22	C27	126.7(6)
C15	Rh1	C12	62.9(3)	C23	C22	Co1	69.0(4)
C15	Rh1	C11	37.2(3)	C23	C22	C27	126.0(6)
C13	Rh1	C5	142.8(2)	C27	C22	Co1	128.1(5)
C13	Rh1	C14	36.4(2)	F3	C6	F1	104.2(5)
C13	Rh1	C11	63.1(2)	F3	C6	C1	113.0(5)
C14	Rh1	C11	62.9(3)	F2	C6	F3	106.1(5)
C12	Rh1	C5	178.4(2)	F2	C6	F1	105.5(5)
C12	Rh1	C13	38.8(2)	F2	C6	C1	115.5(6)
C12	Rh1	C14	62.9(2)	F1	C6	C1	111.5(6)
C12	Rh1	C11	37.1(2)	C1	C5	Rh1	62.1(3)
C31	Co1	C32	41.1(3)	C1	C5	C10	123.3(6)
C31	Co1	C35	40.4(3)	C4	C5	Rh1	105.3(4)
C31	Co1	C21	108.3(3)	C4	C5	C1	109.4(5)
C31	Co1	C25	129.5(3)	C4	C5	C10	123.3(6)
C31	Co1	C34	68.3(3)	C10	C5	Rh1	116.2(5)
C31	Co1	C22	117.1(3)	C25	C24	Co1	69.5(4)
C31	Co1	C24	168.2(3)	C25	C24	C29	126.3(6)
C31	Co1	C23	150.4(3)	C23	C24	Co1	69.7(4)
C33	Co1	C31	68.4(3)	C23	C24	C25	108.3(6)
C33	Co1	C32	40.6(2)	C23	C24	C29	125.3(6)
C33	Co1	C35	68.4(3)	C29	C24	Co1	128.9(5)
C33	Co1	C21	168.9(3)	C3	C2	Rh1	104.7(4)
C33	Co1	C25	149.2(2)	C1	C2	Rh1	63.2(3)
C33	Co1	C34	41.0(3)	C1	C2	C3	108.3(5)
C33	Co1	C22	130.2(3)	C7	C2	Rh1	119.1(5)
C33	Co1	C24	116.5(3)	C7	C2	C3	123.9(6)
C33	Co1	C23	108.3(3)	C7	C2	C1	121.6(5)
C32	Co1	C22	108.4(3)	C14	C15	Rh1	72.0(4)
C35	Co1	C32	68.7(3)	C14	C15	C20	125.5(6)

Table S7. Bond Angles for $[Co(C_5Me_5)_2][Rh(C_5Me_5)(C_5(CF_3)_5)]$.

Atom	Atom	Atom	Angle/°	Atom	Atom	Atom	Angle/°
C35	Co1	C25	107.7(3)	C20	C15	Rh1	125.4(5)
C35	Co1	C22	149.4(3)	C11	C15	Rh1	74.5(4)
C21	Co1	C32	130.1(2)	C11	C15	C14	108.3(6)
C21	Co1	C35	116.4(3)	C11	C15	C20	125.7(6)
C21	Co1	C25	41.0(2)	C14	C13	Rh1	72.3(4)
C21	Co1	C22	40.9(3)	C14	C13	C12	108.2(6)
C21	Co1	C24	69.0(3)	C14	C13	C18	127.2(6)
C25	Co1	C32	168.8(2)	C12	C13	Rh1	70.6(3)
C25	Co1	C22	69.0(3)	C12	C13	C18	124.6(6)
C34	Co1	C32	68.8(3)	C18	C13	Rh1	122.1(5)
C34	Co1	C35	40.5(3)	C3	C4	C5	108.1(5)
C34	Co1	C21	148.9(3)	C3	C4	C9	125.6(6)
C34	Co1	C25	115.9(3)	C5	C4	C9	125.7(6)
C34	Co1	C22	169.1(3)	F8	C8	C3	112.6(6)
C34	Co1	C24	107.7(3)	F9	C8	F8	103.2(6)
C24	Co1	C32	149.2(3)	F9	C8	C3	113.6(6)
C24	Co1	C35	129.5(3)	F7	C8	F8	105.9(6)
C24	Co1	C25	41.1(3)	F7	C8	F9	106.0(6)
C24	Co1	C22	68.9(3)	F7	C8	C3	114.5(6)
C23	Co1	C32	116.9(3)	F6	C7	F4	105.7(5)
C23	Co1	C35	168.0(3)	F6	C7	F5	104.3(5)
C23	Co1	C21	69.0(3)	F6	C7	C2	111.9(5)
C23	Co1	C25	68.8(3)	F4	C7	F5	104.1(5)
C23	Co1	C34	129.6(3)	F4	C7	C2	114.1(5)
C23	Co1	C22	41.3(3)	F5	C7	C2	115.7(5)
C23	Co1	C24	40.5(3)	C15	C14	Rh1	69.2(4)
C32	C31	Co1	69.9(3)	C15	C14	C19	124.7(6)
C32	C31	C36	125.6(6)	C13	C14	Rh1	71.3(4)
C35	C31	Co1	69.9(4)	C13	C14	C15	107.9(6)
C35	C31	C32	108.5(6)	C13	C14	C19	127.4(6)
C35	C31	C36	125.9(7)	C19	C14	Rh1	123.7(5)
C36	C31	Co1	126.6(5)	C22	C23	Co1	69.7(4)
C32	C33	Co1	70.3(3)	C22	C23	C28	124.8(6)
C32	C33	C34	108.7(6)	C24	C23	Co1	69.8(4)
C32	C33	C38	125.1(6)	C24	C23	C22	108.4(6)
C34	C33	Co1	69.7(4)	C24	C23	C28	126.7(6)
C34	C33	C38	126.1(6)	C28	C23	Co1	129.6(5)
C38	C33	Co1	128.7(5)	F14	C10	F13	105.6(5)
C31	C32	Co1	69.0(3)	F14	C10	F15	105.8(6)
C31	C32	C37	126.7(6)	F14	C10	C5	112.8(5)
C33	C32	Co1	69.2(3)	F13	C10	C5	114.6(6)
C33	C32	C31	106.8(6)	F15	C10	F13	104.9(5)
C33	C32	C37	126.5(6)	F15	C10	C5	112.4(6)
C37	C32	Co1	128.3(5)	C13	C12	Rh1	70.6(4)
C31	C35	Co1	69.7(4)	C13	C12	C17	124.9(6)
C31	C35	C34	108.4(6)	C11	C12	Rh1	73.6(4)
C31	C35	C40	126.2(7)	C11	C12	C13	108.0(6)

Table S7. Bond Angles for $[Co(C_5Me_5)_2][Rh(C_5Me_5)(C_5(CF_3)_5)]$.

Atom	Atom	Atom	Angle/˚	Atom	Atom	Atom	Angle/˚
C34	C35	Co1	69.7(4)	C11	C12	C17	126.6(6)
C34	C35	C40	125.3(7)	C17	C12	Rh1	127.3(5)
C40	C35	Co1	129.2(5)	F10	C9	C4	112.4(6)
C25	C21	Co1	69.6(4)	F12	C9	F10	106.2(5)
C25	C21	C26	125.3(6)	F12	C9	F11	107.4(6)
C22	C21	Co1	69.9(4)	F12	C9	C4	114.0(6)
C22	C21	C25	108.4(6)	F11	C9	F10	104.7(6)
C22	C21	C26	126.1(6)	F11	C9	C4	111.6(5)
C26	C21	Co1	130.4(5)	C15	C11	Rh1	68.3(4)
C21	C25	Co1	69.4(4)	C15	C11	C12	107.2(6)
C21	C25	C24	107.7(6)	C15	C11	C16	127.0(7)
C21	C25	C30	125.9(6)	C12	C11	Rh1	69.3(4)
C24	C25	Co1	69.4(4)	C12	C11	C16	125.7(7)
C24	C25	C30	126.4(6)	C16	C11	Rh1	128.5(5)
C30	C25	Co1	127.6(5)				

Table S8. Torsion Angles for $[Co(C_5Me_5)_2][Rh(C_5Me_5)(C_5(CF_3)_5)]$.

A	B	C	D	Angle/˚	A	B	C	D	Angle/˚
Rh1	C1	C6	F3	105.1(7)	C5	C1	C6	F2	-127.4(7)
Rh1	C1	C6	F2	-17.5(10)	C5	C1	C6	F1	112.2(7)
Rh1	C1	C6	F1	-137.9(6)	C5	C1	C2	Rh1	74.1(4)
Rh1	C1	C5	C4	97.5(5)	C5	C1	C2	C3	-23.6(7)
Rh1	C1	C5	C10	-104.5(6)	C5	C1	C2	C7	-176.8(6)
Rh1	C1	C2	C3	-97.6(5)	C5	C4	C9	F10	-158.9(6)
Rh1	C1	C2	C7	109.1(6)	C5	C4	C9	F12	80.1(8)
Rh1	C5	C4	C3	47.9(6)	C5	C4	C9	F11	-41.7(10)
Rh1	C5	C4	C9	-123.7(6)	C2	C3	C4	C5	1.4(7)
Rh1	C5	C10	F14	-40.8(7)	C2	C3	C4	C9	173.1(6)
Rh1	C5	C10	F13	-161.7(4)	C2	C3	C8	F8	-126.8(7)
Rh1	C5	C10	F15	78.6(6)	C2	C3	C8	F9	-9.9(10)
Rh1	C2	C7	F6	57.8(7)	C2	C3	C8	F7	112.1(7)
Rh1	C2	C7	F4	177.7(4)	C2	C1	C6	F3	-147.0(6)
Rh1	C2	C7	F5	-61.5(7)	C2	C1	C6	F2	90.4(8)
Rh1	C15	C14	C13	-61.1(5)	C2	C1	C6	F1	-30.0(9)
Rh1	C15	C14	C19	117.3(7)	C2	C1	C5	Rh1	-72.7(4)
Rh1	C15	C11	C12	58.5(5)	C2	C1	C5	C4	24.8(7)
Rh1	C15	C11	C16	-122.6(7)	C2	C1	C5	C10	-177.2(6)
Rh1	C13	C14	C15	59.8(5)	C13	C12	C11	Rh1	62.6(4)
Rh1	C13	C14	C19	-118.5(7)	C13	C12	C11	C15	4.8(8)
Rh1	C13	C12	C11	-64.6(5)	C13	C12	C11	C16	-174.2(6)
Rh1	C13	C12	C17	122.5(7)	C4	C3	C2	Rh1	-51.3(6)
Rh1	C12	C11	C15	-57.9(5)	C4	C3	C2	C1	14.8(7)
Rh1	C12	C11	C16	123.2(7)	C4	C3	C2	C7	167.3(6)
Co1	C31	C32	C33	59.1(4)	C4	C3	C8	F8	38.1(10)
Co1	C31	C32	C37	-122.9(7)	C4	C3	C8	F9	155.0(7)
Co1	C31	C35	C34	-59.1(5)	C4	C3	C8	F7	-83.0(9)
Co1	C31	C35	C40	124.3(7)	C4	C5	C10	F14	-173.3(6)
Co1	C33	C32	C31	-59.0(4)	C4	C5	C10	F13	65.8(9)
Co1	C33	C32	C37	123.0(7)	C4	C5	C10	F15	-53.8(9)
Co1	C33	C34	C35	59.5(5)	C38	C33	C32	Co1	-124.1(7)
Co1	C33	C34	C39	-122.8(7)	C38	C33	C32	C31	176.9(6)
Co1	C35	C34	C33	-59.2(5)	C38	C33	C32	C37	-1.1(11)
Co1	C35	C34	C39	123.1(7)	C38	C33	C34	Co1	123.8(7)
Co1	C21	C25	C24	59.1(5)	C38	C33	C34	C35	-176.7(6)
Co1	C21	C25	C30	-122.1(7)	C38	C33	C34	C39	1.0(11)
Co1	C21	C22	C23	-58.7(5)	C8	C3	C2	Rh1	116.0(6)
Co1	C21	C22	C27	122.8(7)	C8	C3	C2	C1	-177.8(6)
Co1	C25	C24	C23	59.1(5)	C8	C3	C2	C7	-25.4(10)
Co1	C25	C24	C29	-123.9(7)	C8	C3	C4	C5	-165.5(7)
Co1	C22	C23	C24	-59.3(5)	C8	C3	C4	C9	6.1(11)
Co1	C22	C23	C28	124.8(7)	C30	C25	C24	Co1	122.1(7)
Co1	C24	C23	C22	59.2(5)	C30	C25	C24	C23	-178.7(7)
Co1	C24	C23	C28	-124.9(7)	C30	C25	C24	C29	-1.7(11)
C31	C35	C34	Co1	59.1(5)	C14	C15	C11	Rh1	-64.5(5)
C31	C35	C34	C33	-0.1(8)	C14	C15	C11	C12	-6.0(8)

Table S8. Torsion Angles for [Co(C_5Me_5)$_2$][Rh(C_5Me_5)($C_5(CF_3)_5$)].

A	B	C	D	Angle/°	A	B	C	D	Angle/°
C31	C35	C34	C39	-177.8(7)	C14	C15	C11	C16	172.9(7)
C32	C31	C35	Co1	59.4(4)	C14	C13	C12	Rh1	62.9(5)
C32	C31	C35	C34	0.3(8)	C14	C13	C12	C11	-1.6(8)
C32	C31	C35	C40	-176.3(7)	C14	C13	C12	C17	-174.6(7)
C32	C33	C34	Co1	-59.6(4)	C20	C15	C14	Rh1	-121.1(7)
C32	C33	C34	C35	-0.1(7)	C20	C15	C14	C13	177.8(7)
C32	C33	C34	C39	177.6(7)	C20	C15	C14	C19	-3.8(11)
C35	C31	C32	Co1	-59.5(5)	C20	C15	C11	Rh1	122.7(7)
C35	C31	C32	C33	-0.4(7)	C20	C15	C11	C12	-178.8(7)
C35	C31	C32	C37	177.7(6)	C20	C15	C11	C16	0.1(12)
C21	C25	C24	Co1	-59.1(5)	C10	C5	C4	C3	-175.4(6)
C21	C25	C24	C23	0.0(8)	C10	C5	C4	C9	12.9(11)
C21	C25	C24	C29	177.1(6)	C29	C24	C23	Co1	123.9(7)
C21	C22	C23	Co1	58.9(5)	C29	C24	C23	C22	-176.8(6)
C21	C22	C23	C24	-0.4(8)	C29	C24	C23	C28	-1.0(11)
C21	C22	C23	C28	-176.4(7)	C12	C13	C14	Rh1	-61.8(5)
C25	C21	C22	Co1	59.1(5)	C12	C13	C14	C15	-2.1(8)
C25	C21	C22	C23	0.4(8)	C12	C13	C14	C19	179.6(7)
C25	C21	C22	C27	-178.1(7)	C18	C13	C14	Rh1	117.3(7)
C25	C24	C23	Co1	-59.0(5)	C18	C13	C14	C15	177.1(7)
C25	C24	C23	C22	0.2(8)	C18	C13	C14	C19	-1.2(12)
C25	C24	C23	C28	176.1(7)	C18	C13	C12	Rh1	-116.2(7)
C3	C2	C7	F6	-165.9(6)	C18	C13	C12	C11	179.2(6)
C3	C2	C7	F4	-45.9(9)	C18	C13	C12	C17	6.3(11)
C3	C2	C7	F5	74.9(8)	C36	C31	C32	Co1	121.3(7)
C3	C4	C9	F10	30.9(10)	C36	C31	C32	C33	-179.6(6)
C3	C4	C9	F12	-90.1(9)	C36	C31	C32	C37	-1.6(11)
C3	C4	C9	F11	148.1(7)	C36	C31	C35	Co1	-121.3(7)
C34	C33	C32	Co1	59.3(5)	C36	C31	C35	C34	179.6(6)
C34	C33	C32	C31	0.3(7)	C36	C31	C35	C40	3.0(11)
C34	C33	C32	C37	-177.7(6)	C11	C15	C14	Rh1	66.2(5)
C1	C5	C4	C3	-17.4(8)	C11	C15	C14	C13	5.1(8)
C1	C5	C4	C9	171.0(6)	C11	C15	C14	C19	-176.6(7)
C1	C5	C10	F14	31.6(9)	C26	C21	C25	Co1	125.7(7)
C1	C5	C10	F13	-89.3(8)	C26	C21	C25	C24	-175.2(7)
C1	C5	C10	F15	151.1(6)	C26	C21	C25	C30	3.6(11)
C1	C2	C7	F6	-16.9(9)	C26	C21	C22	Co1	-126.0(7)
C1	C2	C7	F4	103.1(7)	C26	C21	C22	C23	175.3(7)
C1	C2	C7	F5	-136.1(6)	C26	C21	C22	C27	-3.2(11)
C22	C21	C25	Co1	-59.3(5)	C27	C22	C23	Co1	-122.6(7)
C22	C21	C25	C24	-0.3(8)	C27	C22	C23	C24	178.1(6)
C22	C21	C25	C30	178.5(7)	C27	C22	C23	C28	2.1(11)
C6	C1	C5	Rh1	137.4(7)	C17	C12	C11	Rh1	-124.6(7)
C6	C1	C5	C4	-125.1(7)	C17	C12	C11	C15	177.5(7)
C6	C1	C5	C10	32.9(11)	C17	C12	C11	C16	-1.4(11)
C6	C1	C2	Rh1	-136.4(7)	C40	C35	C34	Co1	-124.3(7)
C6	C1	C2	C3	126.0(7)	C40	C35	C34	C33	176.5(7)

Table S8. Torsion Angles for $[Co(C_5Me_5)_2][Rh(C_5Me_5)(C_5(CF_3)_5)]$.

**A**	**B**	**C**	**D**	**Angle/˚**	**A**	**B**	**C**	**D**	**Angle/˚**
C6	C1	C2	C7	-27.2(10)	C40	C35	C34	C39	-1.2(11)
C5	C1	C6	F3	-4.8(10)					

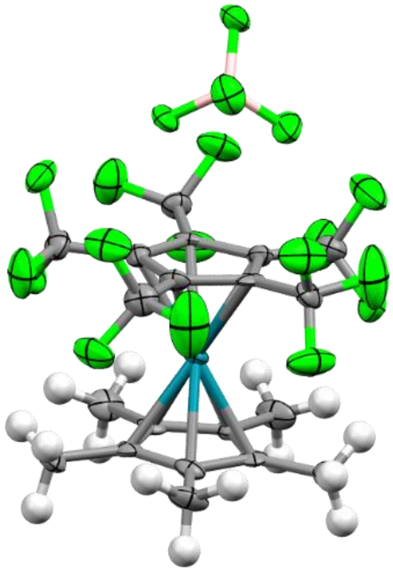

Figure S11. Molecular structure in solid state of [Rh(C_5Me_5)($C_5(CF_3)_5$)][BF_4]. Ellipsoids are depicted with 50% probability level. Color code: grey-carbon; green-fluorine; turquoise-rhodium, boron-yellow.

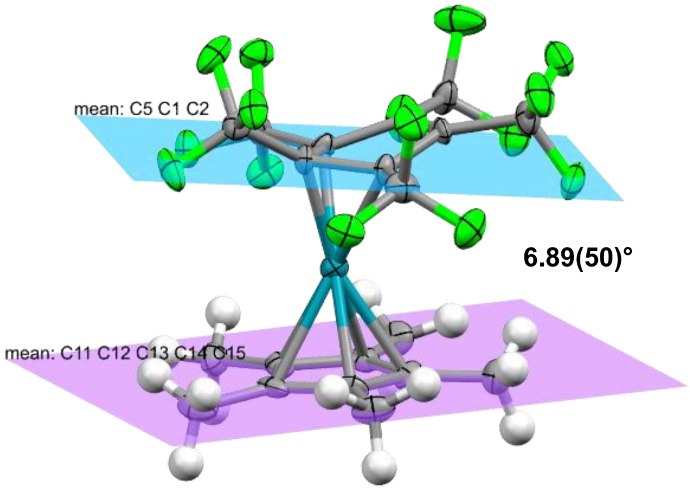

Figure S12. Structure of rhodocene anion [Rh(C_5Me_5)($C_5(CF_3)_5$)]$^-$ in the decamethyl-cobaltocenium salt.

Angle between the allylic bound [$C_5(CF_3)_5$]$^-$ plane (blue) and the plane of the [C_5Me_5]$^-$ coligand plane (purple): 6.89(50)°

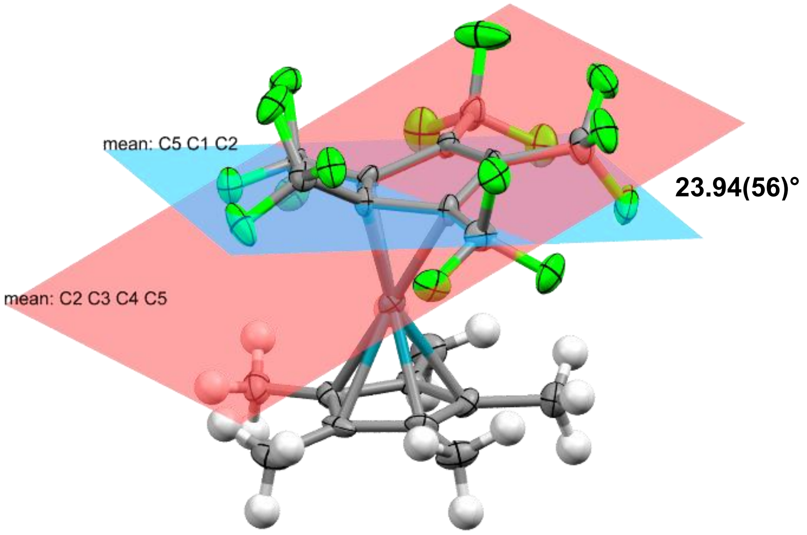

Figure S13. Structure of rhodocene anion $[Rh(C_5Me_5)(C_5(CF_3)_5)]^-$ in the decamethyl-cobaltocenium salt.

Angle between the allylic bound $[C_5(CF_3)_5]^-$ plane (blue) and the plane of the uncoordinated C3−C4 C=C double bond: 23.94(56)°.

Table S9. Selected distances (Å) for [**1**]$^+$ and [**1**]$^-$.

Complex	Rh−Cp$_{centroid}$ for $[C_5Me_5]^-$	Rh−Cp$_{centroid}$ for $[C_5(CF_3)_5]^-$	Rh−Centre$_{C1,C2,C5}$ for $[C_5(CF_3)_5]^-$
[**1**]$^+$	1.8103(8)	1.8540(8)	-
DFT [**1**]$^+$	1.819	1.882	-
[**1**]$^-$	1.8576(7)	-	1.8723(7)
DFT [**1**]$^-$	1.873	-	1.881

EPR Spectra

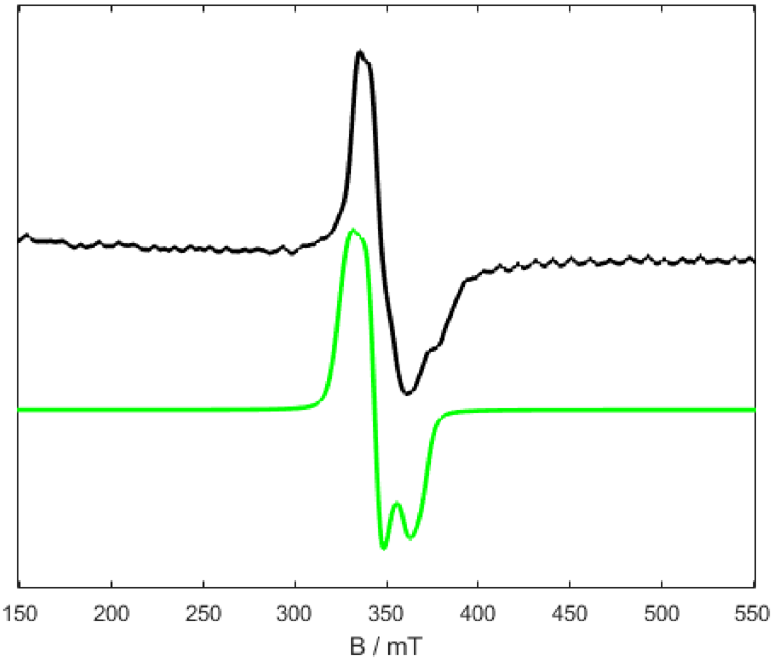

Figure S14. Experimental X-Band EPR spectrum of [**1**] in THF (1.4 mM) at 77 K (black) and simulation (green). The spectrum yields *a* rhombic g-tensor (g_x = 1.877, g_y = 1.998, g_z = 2.085).
A_Rh = [177.4, 10.6, 10.3] MHz

A_F = [46.4, 47.8, 120.3] MHz

The results also agree very well with quantum chemically obtained values (g_x = 1.846, g_y = 1.998, g_z = 2.084).

Results of Quantum Chemical Calculations

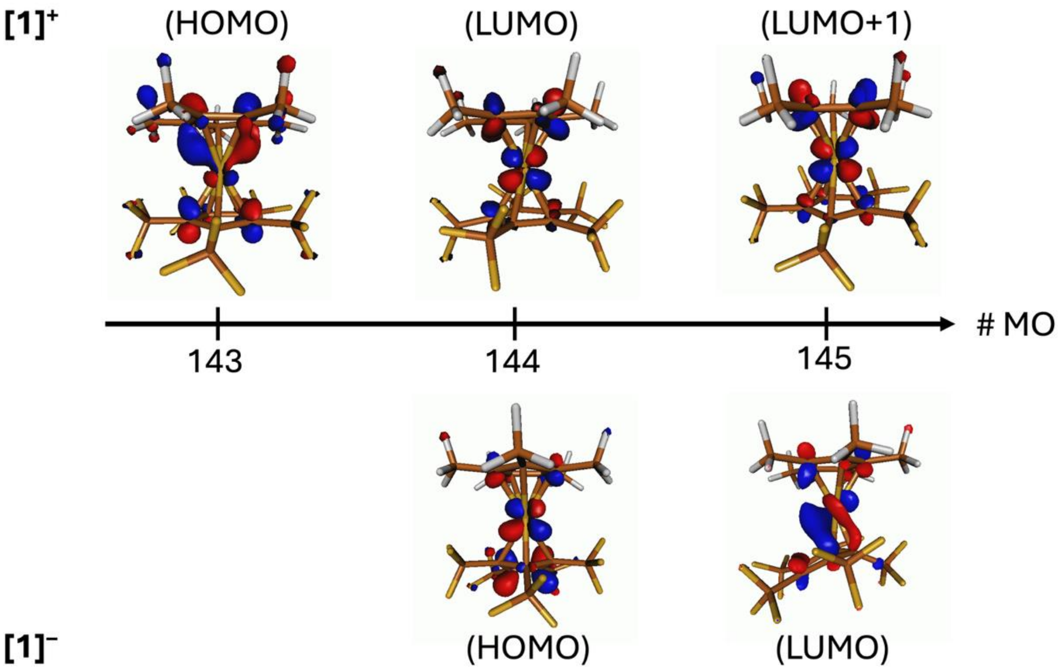

Figure S15. Representation of relevant orbitals of the oxidized and reduced forms of **1** at r^{2}SCAN-3c level. Surfaces are shown for an isovalue of 0.05.

Table S10. Results of the natural population analyses (NPA) and QTAIM analyses performed at the scalar-relativistic BP86-D4/TZ2P//r^{2}SCAN-3c level.

	q^{NPA}	$4d^x$ (Rh)[a]	$5s^x$ (Rh)[b]	q^{QTAIM}	Volume^{QTAIM} [c] [a_0^3]
$[RhCp_2]^+$	0.380	8.32	0.24	0.53	119.1
**[1]**$^+$	0.349	8.38	0.20	0.49	120.8
**[1]**$^-$	0.262	8.42	0.26	0.37	135.2
$[RhCp(CO)_2]$	0.046	8.45	0.44	0.43	135.9

[a] Population of the Rh 4d orbitals based on NPA. [b] Population of the Rh 5s orbital based on NPA. [c] Integrated out to an electron-density iso-surface of 0.002 a.u.

Table S11. Results of energy decomposition analysis (in kcal/mol) for the interaction between the $[C_5(CF_3)_5]^-$ ligand and the $[Rh(I/III)(C_5Me_5)]$ moieties at the BP86-D4/TZ2P//r^{2}SCAN-3c level.

	ΔE_{Pauli}	$\Delta E_{Elstat.}$	$\Delta E_{Orb.Int.}$	ΔE_{COSMO}[a]	ΔE_{Int}
**[1]**$^+$	181.4	−249.7	−165.6	161.0	−94.6
**[1]**$^-$ [b]	288.1	−180.3	−111.8	6.3	−19.4
**[1]**$^-$	245.6	−179.9	−144.7	2.0	−94.9

[a]Contribution only includes the electrostatic interaction energy with the cavity charges. No cavity formation contributions were considered. [b]At the optimized structure of [**1**]$^+$.

Table S12. Sum of the most important NOCV eigenvalues (according to symmetry) for the interaction between the $[C_5(CF_3)_5]^-$ ligand and the $[Rh(I/III)(C_5Me_5)]$ moieties at the BP86-D4/TZ2P//r^{2}SCAN-3c level in kcal/mol.

	σ (bonding)	π (bonding)	π (back-bonding)	δ (back-bonding)
[**1**]$^+$	−24.1	−93.8	---	−21.0
[**1**]$^-$	−19.0	−30.9	−84.6	---

Cartesian Coordinates Optimized at the r^{2}SCAN-3c level

[**1**]$^+$

Rh	9.42994793967677	10.50581411210172	6.13261967795587
F	8.20497214215946	7.87630635571807	3.68072548879224
F	8.70293232966643	8.67816365701500	9.02522278901093
F	6.57952188693803	9.16422420784703	3.01385325181220
F	8.65659478829358	9.81234912114420	2.86922422412593
F	6.61735472093470	9.23053706085169	9.35301539799327
F	7.26499205825504	7.13120744688701	7.62747104104019
F	8.19569526208127	10.72053600324685	9.49882978439848
F	7.78711936212827	13.37781433516282	4.69793918951117
F	8.81914779275228	6.87826170046136	6.13340897679771
F	6.71640406061540	7.01452413286187	5.55021307919433
F	6.35268920809041	13.38272155167704	6.86597818394652
F	7.41007793412102	11.95208472062748	3.11617100365459
F	8.06783432556386	13.08826915919377	8.15575776314578
F	6.17085489775264	12.09833551784381	8.58990832004027
F	5.78872106589424	12.53389949094542	4.41900625469411
C	7.42466707322394	11.13096797858044	5.35007519750183
C	7.72966018285712	9.00756453194440	6.26105676556679
C	7.65255428933407	7.49221116248886	6.39401450846931
C	7.63802175367199	9.94984339144295	7.33886590371156
C	7.65825023205652	9.74480499788280	5.02849899623968
C	11.21558403127893	11.01486795707412	7.31103921820030
C	11.13180106138792	11.29197589099324	4.99164391291534
C	7.40032401086876	11.25236907079596	6.78062531909244
C	7.10736982317635	12.25810147772666	4.38391303484315
C	7.77257590455336	9.14688094495772	3.63574723255840
C	11.04351933171205	11.98370901640247	6.25606991043779
C	11.29382532813887	11.31462203376357	8.76186033403507
H	10.65984685814176	12.15939151305274	9.03731474767040
H	12.33254558425614	11.58214696570011	8.99831051437496
H	11.02351084443781	10.45038265016171	9.37089492913063
C	7.77991683010149	9.63702881516241	8.81688924691296
C	11.34672701626837	9.89414331602181	5.26539172478238
C	11.10744829145603	11.94244416224409	3.65856487532930
H	10.85299169720884	11.24131440253773	2.86336617631950
H	12.11192202562799	12.33725510784810	3.45473425637628
H	10.41074997975058	12.78329847687728	3.63316787478828
C	7.00700300776425	12.47380711787837	7.60084361642364
C	10.91881972575513	13.45178022285448	6.42343369791009

H	10.28677817456300	13.89566563866941	5.65163118773519
H	11.92161790111894	13.88932259613981	6.32365570659333
H	10.53075788707079	13.71943448962974	7.40652250363500
C	11.70136118221090	8.45397880995299	7.40290870904108
H	11.29242151974762	8.44034098010449	8.41411816285764
H	12.79322649960899	8.35977217757849	7.47969038302888
H	11.33058563305647	7.58865875438517	6.85086693812582
C	11.40023090797890	9.72378163044046	6.69938731960946
C	11.57866693136961	8.81837128018748	4.27032168423596
H	11.12544622466204	7.87533780871844	4.58533996189008
H	12.66172514335775	8.65511118609493	4.18966370513698
H	11.20300233730219	9.08507187012017	3.28197131840611

[**1**]^{0}

Rh	9.44863117432110	10.43835158496239	6.10156127692441
F	7.15943749606254	7.93723143463624	3.66051880978415
F	8.83460805447757	9.00298344398806	9.16681638172942
F	6.54580291593594	9.89003767287209	2.96928253277634
F	8.65172362138416	9.33719683526766	2.94215437449904
F	6.66798258093902	9.11389294255816	9.33554122084629
F	8.27027927752760	7.14230243209933	7.61197956669185
F	7.89837332343205	10.90385373951626	9.54113832876770
F	7.72656499417899	13.46147060809003	4.97706433229311
F	8.80596891934948	7.06548460071351	5.51615450787608
F	6.70964834047654	6.97865676424850	6.12636303397729
F	5.75508359634781	13.15979898456350	6.74248866342469
F	8.00289134488263	12.08276980932187	3.32788181389241
F	7.46130124968819	13.27328708261526	8.08585071203523
F	5.75555094428518	12.00692794651830	8.58085458452075
F	5.99651782670277	12.52889216074008	4.03663815933022
C	7.38454637384352	11.17152271029007	5.41684335930633
C	7.79594597865603	9.06366245561882	6.29148892633806
C	7.90352814099561	7.56643901098238	6.39072906303517
C	7.63710548001568	10.02499627242933	7.37183273550574
C	7.63141041442162	9.79372343166165	5.05940085111176
C	11.26066362819393	10.97260999432213	7.28981251835541
C	11.18844176345140	11.22266059653468	4.99109020221868
C	7.12831113447490	11.26148202587311	6.82510359165016
C	7.26898042754871	12.30766814656112	4.43914511807778
C	7.51079075597723	9.23738820628438	3.66125824217036
C	11.12370924855779	11.93488781449241	6.25499184246631
C	11.30915806118923	11.25963808545198	8.75115167096310
H	10.65574103004734	12.09422903443880	9.01877004588533
H	12.33423581280607	11.53288642716004	9.03499962302248
H	11.02228108636174	10.39003292154435	9.34507809793915
C	7.76068155761952	9.75269691318120	8.84768321745474
C	11.48559886577292	9.83922377282172	5.25623947076939
C	11.17092231943060	11.86084940353578	3.64500966742785
H	10.75820379522231	11.19152049569688	2.88670692411880
H	12.19880866177127	12.11089656025318	3.35004646152633
H	10.58920138154888	12.78481042843269	3.64559674525268
C	6.54358201164295	12.41427804461213	7.55351749841704
C	10.97736977619422	13.40799892694644	6.42573486442965
H	10.39431880751544	13.85010340006130	5.61429993550638
H	11.96536506046026	13.88768105543366	6.42445563714145
H	10.48867741099268	13.65306125984963	7.37236708774243
C	11.74155398483859	8.39771117479400	7.40034984913046
H	11.24247958683701	8.36918309981693	8.37106763829222
H	12.82147054654371	8.30749073132300	7.57823515252561

H	11.42419696883966	7.52649295657773	6.82173162609434
C	11.45862153557569	9.66596413657909	6.67151105093609
C	11.77082216897592	8.78555198568548	4.24249920694748
H	11.38721757621176	7.81087444359207	4.55640331618660
H	12.85508491966371	8.68472135480332	4.10035487252220
H	11.33092306780907	9.03273167964571	3.27394959016262

[1]⁻

Rh	9.66769874941975	10.26198255533390	6.20393665675155
F	8.48432118794586	7.94754773652201	3.99562167750759
F	8.72160752622912	9.49616801164326	9.18690046540398
F	6.68651212854812	8.90262587179788	3.21401147235331
F	8.64913565312353	9.75438848205001	2.81997717865195
F	6.63444668170600	10.05064158008036	9.39686055535003
F	7.13137962231655	7.70880984264870	7.97062777073481
F	8.15971197929639	11.59804918367248	9.29192394897323
F	6.65376832391877	13.30568199979218	4.27437428206177
F	8.59419899130027	6.97445928975055	6.55269291887045
F	6.56364701892574	7.40779681173742	5.89752695060540
F	5.18989070109823	13.10946732321843	6.42890972173524
F	6.92455520616231	11.65803467497539	2.90411918554860
F	7.09756526857869	13.66688404432614	7.32631085723053
F	5.71347403806509	12.36680441616425	8.38825088682480
F	5.04407171296536	11.86305452987865	3.96293454107594
C	6.94269068570535	11.13803116608420	5.21441664682929
C	7.94651996088112	9.29079102886410	6.38715515198383
C	7.57582686494642	7.86399155112730	6.69752274608131
C	7.71662373787567	10.45706594405713	7.27379383905349
C	7.80637344563467	9.94257044274196	5.05702820172467
C	11.50944793578883	10.60860476565854	7.37549247889913
C	11.36324567940041	11.28413520983999	5.16815963965274
C	6.87213139784241	11.43344878448708	6.54662103352515
C	6.39678788095362	11.97711944210259	4.10695660764917
C	7.90476762325402	9.15879401642654	3.79754082459304
C	11.32309174133831	11.75960243007602	6.49339227581671
C	11.63182257828977	10.69802815245146	8.86009051505877
H	10.91601537713014	11.41080227128122	9.27864065287980
H	12.63928137002249	11.04054528323403	9.13579199194560
H	11.46362664544835	9.72965659899078	9.33714863264126
C	7.79999217069883	10.39175677611127	8.75279065931814
C	11.59933082932368	9.83640704706857	5.21215295061505
C	11.25795458003061	12.08999043858319	3.91474541001613
H	10.70962439972403	11.54805217435090	3.13897019663845
H	12.25579579242746	12.31755375789135	3.51435203519969
H	10.74320483040249	13.03835314533378	4.09149546033116
C	6.22421714797799	12.62500176244999	7.15802956820164
C	11.16715082964635	13.17292144561944	6.95304810693846
H	10.68632750058851	13.79109516445807	6.18977768008610
H	12.14336406627178	13.62300843909869	7.18294397559817
H	10.55944427900752	13.23053590715934	7.86141773674905
C	12.13615266667266	8.09234659300121	7.07585967688674
H	11.68525689203221	7.89000258984072	8.05181744518777
H	13.22526346595807	7.99706925488432	7.18848913745177
H	11.80593628932973	7.31001802339335	6.38656678501355
C	11.76575134106093	9.45043657485051	6.57485048326878
C	11.82945599077380	8.97432097452364	4.01525252037820
H	11.58330721914056	7.92885333924162	4.22178538131072
H	12.88384423889170	9.01587879548856	3.70684128256477
H	11.22470275592925	9.30162135563641	3.16582720023220

Cyclic Voltammetry

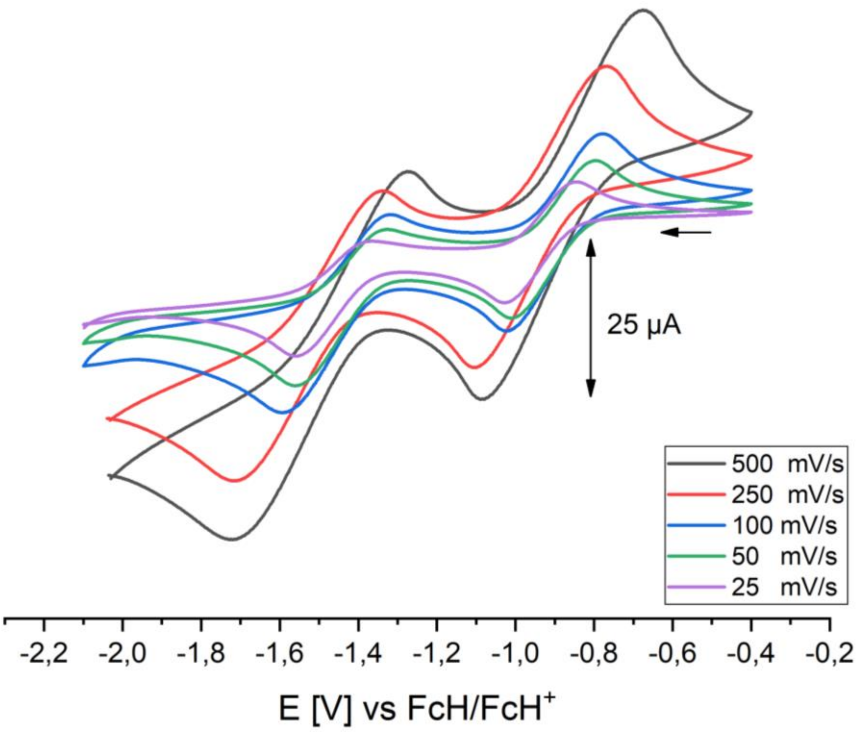

Figure S16. Cyclic voltammogram (current in µA versus potential in V vs. $FcH^{0/+}$).

Table S13. Electrochemical parameters

Scan rates	I_{pa}/I_{pc} (E_{red2})	I_{pa}/I_{pc} (E_{red1})	ΔE (E_{red2})	ΔE (E_{red1})
25 mV/s	1.73	1.28	0.185 V	0.179 V
50 mV/s	1.55	1.32	0.232 V	0.214 V
100 mV/s	1.41	1.26	0.273 V	0.241 V
250 mV/s	0.99	1.20	0.371 V	0.340 V
500 mV/s	0.97	1.18	0.440 V	0.410 V

References

(1) (a) Fulmer, G. R.; Miller, A. J. M.; Sherden, N. H.; Gottlieb, H. E.; Nudelman, A.; Stoltz, B. M.; Bercaw, J. E.; Goldberg, K. I. NMR Chemical Shifts of Trace Impurities: Common Laboratory Solvents, Organics, and Gases in Deuterated Solvents Relevant to the Organometallic Chemist. *Organometallics* **2010**, *29*, 2176−2179. (b) Gottlieb, H. E.; Kotlyar, V.; Nudelman, A. NMR Chemical Shifts of Common Laboratory Solvents as Trace Impurities. *J. Org. Chem.* **1997**, *62*, 7512−7515.
(2) Harris, R. K.; Becker, E. D.; De Menezes, S. M.; Granger, P.; Hoffman, R. E.; Zilm, K. W. Further conventions for NMR shielding and chemical shifts (IUPAC Recommendations 2008). *Magn. Reson. Chem.* **2008**, *46*, 582−598.
(3) Willcott, M. R. MestRe Nova. *J. Am. Chem. Soc.* **2009**, *131*, 13180−13180.
(4) Dolomanov, O. V.; Bourhis, L. J.; Gildea, R. J.; Howard, J. A. K.; Puschmann, H. OLEX2: a complete structure solution, refinement and analysis program. *J. Appl. Crystallogr.* **2009**, *42*, 339−341.
(5) Sheldrick, G. SHELXT - Integrated space-group and crystal-structure determination. *Acta Crystallogr.* **2015**, *A71*, 3−8.
(6) Sheldrick, G. A short history of SHELX. *Acta Crystallogr.* **2008**, *A64*, 112-122.

(7) Macrae, C. F.; Edgington, P. R.; McCabe, P.; Pidcock, E.; Shields, G. P.; Taylor, R.; Towler, M.; van de Streek, J. Mercury: visualization and analysis of crystal structures. *J. Appl. Crystallogr.* **2006**, *39*, 453−457.
(8) *Persistence of Vision Pty. Ltd. Persistence of Vision Raytracer. Ltd., Persistence of Vision Pty. 2004. Retrieved from http://www.povray.org/download/.*
(9) Westrip, S. publCIF: software for editing, validating and formatting crystallographic information files. *J. Appl. Cryst.* **2010**, *43*, 920−925.
(10) Grimme, S.; Hansen, A.; Ehlert, S.; Mewes, J.-M. r2SCAN-3c: A "Swiss army knife" composite electronic-structure method. *J. Chem. Phys.* **2021**, *154*, 064103.
(11) Neese, F. Software update: The ORCA program system—Version 5.0. *WIREs Comput. Mol. Sci.* **2022**, *12*, e1606.
(12) te Velde, G.; Bickelhaupt, F. M.; Baerends, E. J.; Fonseca Guerra, C.; van Gisbergen, S. J. A.; Snijders, J. G.; Ziegler, T. Chemistry with ADF. *J. Comp. Chem.* **2001**, *22*, 931−967.
(13) Adamo, C.; Barone, V. Toward reliable density functional methods without adjustable parameters: The PBE0 model. *J. Chem. Phys.* **1999**, *110*, 6158−6170.
(14) Bühl, M. Density functional computations of transition metal NMR chemical shifts: dramatic effects of Hartree-Fock exchange. *Chem. Phys. Lett.* **1997**, *267* , 251−257.
(15) Autschbach, J. The role of the exchange-correlation response kernel and scaling corrections in relativistic density functional nuclear magnetic shielding calculations with the zeroth-order regular approximation. *Mol. Phys.* **2013**, *111*, 2544−2554.
(16) Ziegler, T.; Rauk, A. On the calculation of bonding energies by the Hartree Fock Slater method. *Theor. Chim. Acta* **1977**, *46*, 1−10.
(17) Mitoraj, M. P.; Michalak, A.; Ziegler, T. On the Nature of the Agostic Bond between Metal Centers and β-Hydrogen Atoms in Alkyl Complexes. An Analysis Based on the Extended Transition State Method and the Natural Orbitals for Chemical Valence Scheme (ETS-NOCV). *Organometallics* **2009**, *28*, 3727−3733.
(18) (a) Becke, A. D. Density-functional exchange-energy approximation with correct asymptotic behavior. *Phys. Rev. A* **1988**, *38*, 3098−3100. (b) Perdew, J. P. Density-functional approximation for the correlation energy of the inhomogeneous electron gas. *Phys. Rev. B* **1986**, *33*, 8822−8824. (c) Perdew, J. P. Erratum: Density-functional approximation for the correlation energy of the inhomogeneous electron gas. *Phys. Rev. B* **1986**, *34*, 7406−7406. (d) Caldeweyher, E.; Ehlert, S.; Hansen, A.; Neugebauer, H.; Spicher, S.; Bannwarth, C.; Grimme, S. A generally applicable atomic-charge dependent London dispersion correction. *J. Chem. Phys.* **2019**, *150*, 154122.
(19) Reed, A. E.; Weinstock, R. B.; Weinhold, F. Natural population analysis. *J. Chem. Phys.* **1985**, *83*, 735−746.
(20) (a) Bader, R. F. W. Principle of stationary action and the definition of a proper open system. *Phy. Rev. B* **1994**, *49*, 13348−13356. (b) Rodríguez, J. I.; Köster, A. M.; Ayers, P. W.; Santos-Valle, A.; Vela, A.; Merino, G. An efficient grid-based scheme to compute QTAIM atomic properties without explicit calculation of zero-flux surfaces. *J. Comput. Chem.* **2009**, *30*, 1082−1092. (c) Rodríguez, J. I.; Bader, R. F. W.; Ayers, P. W.; Michel, C.; Götz, A. W.; Bo, C. A high performance grid-based algorithm for computing QTAIM properties. *Chem. Phys. Lett.* **2009**, *472*, 149−152.
(21) OriginPro, 2017G. OriginLab Corporation, Northampton, MA, USA.
(22) Stoll, S.; Schweiger, A. EasySpin, a comprehensive software package for spectral simulation and analysis in EPR. *J. Magn. Reson.* **2006**, *178*, 42−55.
